# Body weight and composition endpoints in cancer cachexia clinical trials: Systematic Review 4 of the cachexia endpoints series

**DOI:** 10.1002/jcsm.13478

**Published:** 2024-05-13

**Authors:** Leo R. Brown, Mariana S. Sousa, Michael S. Yule, Vickie E. Baracos, Donald C. McMillan, Jann Arends, Trude R. Balstad, Asta Bye, Olav Dajani, Ross D. Dolan, Marie T. Fallon, Christine Greil, Marianne J. Hjermstad, Gunnhild Jakobsen, Matthew Maddocks, James McDonald, Inger O. Ottestad, Iain Phillips, Judith Sayers, Melanie R. Simpson, Ola M. Vagnildhaug, Tora S. Solheim, Barry J.A. Laird, Richard J.E. Skipworth, the Cancer Cachexia Endpoints Working Group

**Affiliations:** ^1^ Clinical Surgery The University of Edinburgh, Royal Infirmary of Edinburgh Edinburgh UK; ^2^ Improving Palliative, Aged and Chronic Care Through Clinical Research and Translation (IMPACCT) University of Technology Sydney Sydney Australia; ^3^ Institute of Genetics and Cancer The University of Edinburgh, Western General Hospital Edinburgh UK; ^4^ St Columba's Hospice Care Edinburgh UK; ^5^ Department of Oncology University of Alberta Edmonton Alberta Canada; ^6^ Academic Unit of Surgery University of Glasgow, Glasgow Royal Infirmary Glasgow UK; ^7^ Department of Medicine I, Medical Centre—University of Freiburg Faculty of Medicine University of Freiburg Freiburg Germany; ^8^ Department of Clinical and Molecular Medicine, Faculty of Medicine and Health Sciences Norwegian University of Science and Technology Trondheim Norway; ^9^ Department of Clinical Medicine, Clinical Nutrition Research Group UiT The Arctic University of Norway Tromsø Norway; ^10^ Department of Oncology Oslo University Hospital Oslo Norway; ^11^ Department of Nursing and Health Promotion, Faculty of Health Sciences Oslo Metropolitan University Oslo Norway; ^12^ Department of Public Health and Nursing, Faculty of Medicine and Health Sciences Norwegian University of Science and Technology Trondheim Norway; ^13^ Cancer Clinic St. Olav's Hospital, Trondheim University Hospital Trondheim Norway; ^14^ Cicely Saunders Institute of Palliative Care, Policy and Rehabilitation King's College London London UK; ^15^ Department of Nutrition, Institute of Basic Medical Sciences University of Oslo Oslo Norway; ^16^ The Clinical Nutrition Outpatient Clinic, Section of Clinical Nutrition, Department of Clinical Service, Division of Cancer Medicine Oslo University Hospital Oslo Norway; ^17^ Edinburgh Cancer Centre Western General Hospital Edinburgh UK

**Keywords:** body composition, cachexia, cancer cachexia, clinical trials

## Abstract

Significant variation exists in the outcomes used in cancer cachexia trials, including measures of body composition, which are often selected as primary or secondary endpoints. To date, there has been no review of the most commonly selected measures or their potential sensitivity to detect changes resulting from the interventions being examined. The aim of this systematic review is to assess the frequency and diversity of body composition measures that have been used in cancer cachexia trials. MEDLINE, Embase and Cochrane Library databases were systematically searched between January 1990 and June 2021. Eligible trials examined adults (≥18 years) who had received an intervention aiming to treat or attenuate the effects of cancer cachexia for >14 days. Trials were also of a prospective controlled design and included body weight or at least one anthropometric, bioelectrical or radiological endpoint pertaining to body composition, irrespective of the modality of intervention (e.g., pharmacological, nutritional, physical exercise and behavioural) or comparator. Trials with a sample size of <40 patients were excluded. Data extraction used Covidence software, and reporting followed the Preferred Reporting Items for Systematic Reviews and Meta‐Analyses guidance. This review was prospectively registered (PROSPERO: CRD42022276710). A total of 84 clinical trials, comprising 13 016 patients, were eligible for inclusion. Non‐small‐cell lung cancer and pancreatic cancer were studied most frequently. The majority of trial interventions were pharmacological (52%) or nutritional (34%) in nature. The most frequently reported endpoints were assessments of body weight (68 trials, *n* = 11 561) followed by bioimpedance analysis (BIA)‐based estimates (23 trials, *n* = 3140). Sixteen trials (*n* = 3052) included dual‐energy X‐ray absorptiometry (DEXA)‐based endpoints, and computed tomography (CT) body composition was included in eight trials (*n* = 841). Discrepancies were evident when comparing the efficacy of interventions using BIA‐based estimates of lean tissue mass against radiological assessment modalities. Body weight, BIA and DEXA‐based endpoints have been most frequently used in cancer cachexia trials. Although the optimal endpoints cannot be determined from this review, body weight, alongside measurements from radiological body composition analysis, would seem appropriate. The choice of radiological modality is likely to be dependent on the trial setting, population and intervention in question. CT and magnetic resonance imaging, which have the ability to accurately discriminate tissue types, are likely to be more sensitive and provide greater detail. Endpoints are of particular importance when aligned with the intervention's mechanism of action and/or intended patient benefit.

## Introduction

Cancer cachexia is a complex multifactorial syndrome characterized by loss of muscle and body fat.^1^ These changes are strongly associated with poorer quality of life, increased morbidity and worse survival.^2^ The 2011 consensus definition for cancer cachexia provided diagnostic and staging criteria that have been instrumental in aiding cachexia trial design.^1^ At present, there is no similar consensus regarding endpoints, and significant variations remain amongst the clinical assessments used in cancer cachexia trials.

A comprehensive patient assessment of cachexia would consider changes in body composition, dietary intake, biomarkers of the pathophysiological drivers of cachexia, physical function and quality of life, and the influence on associated oncological outcomes. Depending on the mechanism of a given clinical trial intervention, particular weighting may be assigned to chosen measures within this broad range. Selected endpoints must be both sensitive enough to detect change and specific enough not to be readily influenced by other conditions or treatments. Furthermore, it is imperative that they convey clinical relevance.

Endpoints pertaining to body weight and composition are amongst the most frequently reported in cachexia trials and will be the focus of this review. Anthropometric measurements and electric bioimpedance analysis (BIA) are simple modalities that, although inexpensive and non‐invasive, are prone to confounders and provide finite levels of detail. Dual‐energy X‐ray absorptiometry (DEXA) is widely available and can provide estimates of regional/whole‐body fat or lean tissue mass. However, DEXA is unable to discriminate between different types of ‘lean tissue’ (e.g., skeletal muscle vs. organs) or anatomical locations (e.g., visceral vs. subcutaneous adipose tissue).^3^ While cachexia research has traditionally focused on the loss of muscle, it is now known that adipose tissue also plays an important role in cachexia pathophysiology, and different mechanisms underpin the loss of each tissue type.^4^ As such, the ability of modalities to distinguish between body tissue compartments is of increasing relevance. Computed tomography (CT) and magnetic resonance imaging (MRI) scans are considered the ‘gold‐standard’ assessment modalities for body composition owing to their specificity in discriminating tissue identities and their precision.^5^ Comparison of the two has shown high levels of agreement in assessments of muscle quantity and quality^6^; however, CT has been more frequently utilized in cachexia research owing to its more widespread use in routine clinical practice.

At present, it is not known what the best endpoints for cancer cachexia trials are. This may have resulted in sub‐optimal clinical trial design, which could have in turn hindered the development of effective therapies. An appraisal of the endpoints currently used would seem like a logical starting point. The aim of this systematic review is to assess the frequency and diversity of measures that have been used to assess body weight and body composition in cancer cachexia trials.

## Methods

This systematic review is reported in accordance with the Preferred Reporting Items for Systematic Reviews and Meta‐Analyses (PRISMA) statement.^7^ The review protocol was prospectively registered at the International Prospective Register of Systematic Reviews: PROSPERO (CRD42022276710).^8^

This review will address assessments of body weight, alongside anthropometric, bioelectrical or radiological endpoints pertaining to body composition. It is one of a series of six that will comprehensively evaluate the endpoints examined in cancer cachexia trials. Given the breadth of outcome measures in the literature, these were categorized broadly under the following domains: physical function,^9^ quality of life, appetite and dietary intake, body weight and composition, oncological outcomes and biomarkers.

### Search strategy

A systematic search of MEDLINE (Ovid), Embase (Ovid) and Cochrane Central Register of Controlled Trials databases was conducted by a senior research librarian (University of Oslo). All published studies from 1 January 1990 to 2 June 2021 were eligible. Search results were synthesized and managed using the web‐based systematic review software ‘Covidence’ (Veritas Health Innovations, Melbourne, Australia), and duplicates were removed. A detailed search strategy is outlined in *Appendix* *A*.

### Study eligibility criteria

Prospective clinical trials that considered an intervention aiming to treat or attenuate the effects of cachexia in adult patients (≥18 years) with cancer were considered for eligibility. Inclusion was irrespective of the site of primary malignancy, modality of intervention (e.g., pharmacological, nutritional and physical exercise) or choice of comparator. Articles were excluded if they studied fewer than 40 patients and/or if the intervention lasted <14 days. Studies in which patients underwent surgery during the assessment period were excluded. All included full‐text articles were written in the English language.

### Data selection and extraction

The titles and abstracts of the identified studies were independently reviewed by three authors (OD, TSS and BJAL). Those selected were subsequently subject to full‐text review (LRB and MSS). In instances of discrepancies between reviewers regarding an article's inclusion, consensus was reached through consultation between reviewers or with the wider authorship group. A pre‐defined data extraction table was developed and pilot‐tested before relevant data points were extracted independently by the lead authors (LRB and MSS).

### Relevant outcome measures

Endpoints considered by this review were those pertaining to assessments of body weight and other modalities that aim to assess changes in body composition. These shall be categorized as anthropometric (e.g., body weight, circumference or skinfold measurements), bioelectrical (e.g., BIA) or radiological (e.g., DEXA, CT or MRI) measure of body composition.

### Assessment of methodology and risk of bias

The methodological quality of each study was independently assessed by four reviewers (JS, JM, OD and BJAL) using the modified Downs and Black checklist.^10^ This tool assesses several criteria including study design, internal and external validity, and reporting standards.

### Data analysis

Study characteristics, patient details and disease demographics were reported descriptively. The aim of this review was to describe the body weight and composition outcomes used, rather than estimate treatment effects. As such, quantitative meta‐analysis was not performed. Furthermore, the heterogenous nature of the trials and interventions studied made meta‐analysis of treatment effects on each endpoint impractical. Analyses and visualization were conducted using RStudio Version 4.2.2 (R Foundation for Statistical Computing, Vienna, Austria) with packages including *maps* and *tidyverse*.

## Results

**Figure 1:**
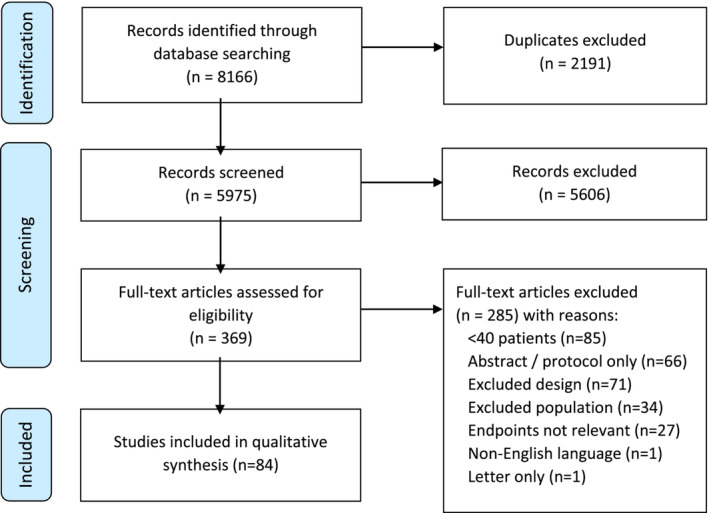
PRISMA flow chart.

Overall, 8166 studies were identified following systematic searches of MEDLINE (Ovid), Embase (Ovid) and Cochrane Central Register of Controlled Trials databases (*Appendix* *A*). Following the removal of duplicates (*n* = 2191), further screening of the title and abstract for 5975 studies was performed. Of these, 5606 articles were excluded and 369 were retrieved for full‐text review. Following detailed screening against the chosen inclusion and exclusion criteria, 84 clinical trials were eligible for inclusion. The PRISMA flow chart is detailed in *Figure* *1*.

### Study characteristics

Between 1990 and 2021, a total of 84 prospective clinical trials (*n* = 13 016 participants) included body weight or measure(s) of body composition as an endpoint. While numerous primary tumour sites were considered, pancreatic cancer (*n* = 11 trials) and non‐small‐cell lung cancer (*n* = 10 trials) were the most frequently studied. Cohorts ranged in size, with the largest cohort being 979 patients studied by the ROMANA 1 and 2 trials.^11^ Pharmacological interventions (*n* = 43 trials) were most evaluated, followed by nutritional (*n* = 28 trials), multi‐modal (*n* = 9 trials) and exercise‐based modalities (*n* = 4 trials). The key characteristics of the included trials are detailed in *Table* *1*.

**Table 1:** Key characteristics of included clinical trials

Author (reference)	Year	Sample size	Study design	Study quality	Primary cancer site	Intervention	Comparator	Body composition outcomes^a^
Kardinal et al.^12^	1990	293	RCT	8	Any malignancy (not brain)	Cyproheptadine (**pharmacological**)	Placebo	Body weight^c^ (*primary*)
Loprinzi et al.^13^	1990	133	RCT	9	Any malignancy (not brain/breast/endometrial)	Megestrol acetate (**pharmacological**)	Placebo	**Body weight** ^b^ ^,^ ^c^ (*primary*)
Feliu et al.^14^	1992	150	RCT	5	Any malignancy (not hormone dependent)	Megestrol acetate (**pharmacological**)	Placebo	Body weight^b^ (*primary*)
Downer et al.^15^	1993	60	RCT	1	Any malignancy	Medroxyprogesterone acetate (**pharmacological**)	Placebo	Body weight^b^ (*primary*) Mid‐arm circumference Triceps skinfold thickness
Loprinzi et al.^16^	1993	342	Phase III RCT	8	Any malignancy (not breast/endometrial)	Megestrol acetate 1280 mg or megestrol acetate 800 mg (**pharmacological**)	Megestrol acetate 480 mg or megestrol acetate 160 mg	Body weight^c^ (*primary*)
Ovesen et al.^17^	1993	105	RCT	8	Small‐cell‐lung/ovarian/breast	Nutritional counselling (**nutritional**)	Standard care	Body weight^b^ (*primary*) Arm muscle area **Triceps skinfold thickness**
Goldberg et al.^18^	1995	70	RCT	8	Any malignancy (not primary brain tumour)	Pentoxifylline (**pharmacological**)	Placebo	Body weight^c^ (*primary*)
Gebbia et al.^19^	1996	122	RCT	6	Any malignancy (not hormone dependent)	Megestrol acetate 320 mg (**pharmacological**)	Megestrol acetate 160 mg	Body weight (*primary*)
Lissoni et al.^20^	1996	100	RCT	7	Any solid tumour	Melatonin (**pharmacological**)	Standard care	**Body weight** ^b^ (*primary*)
Simons et al.^21^	1996	206	RCT	7	Any malignancy (not hormone dependent)	Medroxyprogesterone acetate (**pharmacological**)	Placebo	**Body weight** ^b^ (*primary*)
Beller et al.^22^	1997	240	RCT	4	Any malignancy (not hormone dependent)	Megestrol acetate 480 mg or megestrol acetate 160 mg (**pharmacological**)	Placebo	Body weight^b^ (*primary*) Mid‐arm circumference Mid‐arm fat and muscle area Triceps skinfold thickness
Chen et al.^23^	1997	129	RCT	8	Head and neck	Megestrol acetate or prepulside (**pharmacological**)	Placebo	**Body weight** ^b^ (*primary*)
Daneryd et al.^24^	1998	180	RCT	7	Any malignancy	Indomethacin + erythropoietin (**pharmacological**)	Indomethacin	Lean body mass—DEXA **Body weight** ^b^
De Conno et al.^25^	1998	42	RCT	6	Any malignancy (not hormone dependent)	Megestrol acetate (**pharmacological**)	Placebo	**Body weight** ^b^ (*primary*)
Vadell et al.^26^	1998	150	RCT	5	Any malignancy	Megestrol acetate 480 mg or megestrol acetate 160 mg (**pharmacological**)	Placebo	**Body weight** ^b^ (*primary*) Mid‐arm circumference **Triceps skinfold thickness**
Loprinizi et al.^27^	1999	496	RCT	8	Any malignancy (not breast/prostate/ovarian/endometrial)	Megestrol acetate or dexamethasone (**pharmacological**)	Fluoxymesterone	Body weight^c^ (*primary*)
McMillan et al.^28^	1999	73	RCT	7	Gastrointestinal	Megestrol acetate + ibuprofen (**pharmacological**)	Megestrol acetate + placebo	**Body weight** ^b^ (*primary*) **Mid‐arm circumference** Triceps skinfold thickness Biceps skinfold thickness
Westman et al.^29^	1999	255	RCT	7	Other mixed	Megestrol acetate (**pharmacological**)	Placebo	Body weight^b^ (*primary*)
Jatoi et al.^30^	2002	469	RCT	10	Any malignancy (not brain/breast/ovarian/endometrial)	Megestrol acetate + dronabinol or megestrol acetate + placebo (**pharmacological**)	Dronabinol + placebo	**Body weight** ^c^ (*primary*)
Persson et al.^31^	2002	144	RCT	6	Breast/colorectal/gastric/prostate	Individual nutritional counselling or individual and group nutritional counselling (**nutritional**)	Group nutritional counselling or standard care	**Body weight** ^c^ (*primary*)
Ulutin et al.^32^	2002	119	RCT	9	NSCLC	Megestrol acetate 320 mg (**pharmacological**)	Megestrol acetate 160 mg	**Body weight** (increase vs. stable vs. decrease)
Bruera et al.^33^	2003	91	RCT	7	Any malignancy	Fish oil capsules (**nutritional**)	Placebo	Lean body mass—BIA Body weight^b^ Mid‐arm muscle circumference Triceps skinfold thickness Subscapular skinfold thickness
Fearon et al.^34^	2003	200	RCT	8	Pancreatic	*n*‐3 fatty acid and antioxidant‐enriched supplement (**nutritional**)	Supplement without *n*‐3 fatty acid and antioxidants	Lean body mass—BIA Body weight^b^
Isenring et al.^35^	2004	60	RCT	8	Gastrointestinal/head and neck	Nutrition counselling and protocol (**nutritional**)	Standard care	Fat‐free mass—BIA **Body weight** ^b^
Lundholm et al.^36^	2004	309	RCT	5	Any solid tumour	Indomethacin + erythropoietin + nutritional support + home total parenteral nutrition (**multi‐modal**)	Indomethacin + erythropoietin	Fat mass—DEXA Lean body mass—DEXA Body weight^b^ Mid‐arm muscle circumference Triceps skinfold thickness
Gonçalves Dias et al.^37^	2005	64	Non‐randomized trial	1	Head and neck	Home enteral (nasogastric) feeding or oral diet + nutritional supplements (**nutritional**)	Oral diet	Body weight^b^ BMI Mid‐arm circumference Mid‐arm muscle area Triceps skinfold thickness
Gordon et al.^38^	2005	50	RCT	10	Pancreatic	Thalidomide (**pharmacological**)	Placebo	**Body weight** ^b^ (*primary*) **Bone‐free arm muscle area**
Fearon et al.^39^	2006	518	RCT	8	Gastrointestinal/lung	EPA 2 g or EPA 4 g (**pharmacological**)	Placebo	Lean body mass—BIA Body weight^b^
Berk et al.^40^	2008	472	RCT	9	Any solid tumour	Nutritional supplement (**nutritional**)	Placebo	Lean body mass—BIA (*primary*) Body weight^c^ Various skinfold thickness
Wiedenmann et al.^41^	2008	86	Phase II RCT	7	Pancreatic	Infliximab 5 mg/kg or infliximab 3 mg/kg (**pharmacological**)	Placebo	Lean body mass—BIA (*primary*)
Beijer et al.^42^	2009	100	RCT	8	Any malignancy	Adenosine 5′‐triphosphate (**pharmacological**)	Standard care	**Triceps skinfold thickness** (*primary*) Body weight^b^ Mid‐arm circumference
Mantovani et al.^43^	2010	332	Phase III RCT	7	Any malignancy	Megestrol acetate or EPA‐enriched nutritional supplement or l‐carnitine or thalidomide (**pharmacological**)	Megestrol acetate + EPA‐enriched nutritional supplement + l‐carnitine + thalidomide	**Lean body mass—DEXA** (*primary*) Lean body mass—BIA (*primary*) **Lean body mass—CT** (*primary*)
Navari et al.^44^	2010	80	RCT	7	Gastrointestinal/lung	Megestrol acetate + olanzapine (**pharmacological**)	Megestrol acetate	**Body weight** ^c^ (*primary*)
Baldwin et al.^45^	2011	358	RCT	8	Gastrointestinal/NSCLC/mesothelioma	Nutritional supplement + nutritional counselling or nutritional supplement (**nutritional**)	Nutritional counselling or standard care	Body weight^b^
Kraft et al.^46^	2012	72	RCT	10	Pancreatic	l‐Carnitine supplement (**nutritional**)	Placebo	**Fat mass—BIA** **BMI**
Macciò et al.^47^	2012	144	Phase III RCT	8	Gynaecological	Megestrol acetate + l‐carnitine + celecoxib + antioxidants (**pharmacological**)	Megestrol acetate	**Lean body mass—DEXA** (*primary*)
Madeddu et al.^48^	2012	60	Phase III RCT	7	Any malignancy	l‐Carnitine + celecoxib + megestrol acetate (**pharmacological**)	l‐Carnitine + celecoxib	Lean body mass—DEXA (*primary*) Lean body mass—CT (*primary*) Lean body mass—BIA (*primary*)
Silander et al.^49^	2012	134	RCT	6	Head and neck	Prophylactic PEG (**nutritional**)	Standard care	Body weight^c^ (*primary*) BMI
Wen et al.^50^	2012	102	RCT	5	Any malignancy	Megestrol acetate + thalidomide (**pharmacological**)	Megestrol acetate	**Body weight** ^b^ (*primary*)
Del Fabbro et al.^51^	2013	73	RCT	10	Gastrointestinal/lung	Melatonin (**pharmacological**)	Placebo	Body weight^b^ (*primary*) Lean body mass—BIA Fat‐free mass—BIA
Dobs et al.^52^	2013	159	Phase II RCT	8	Other mixed	Enobosarm 1 mg or enobosarm 3 mg (**pharmacological**)	Placebo	**Lean body mass—DEXA** (*primary*) Body weight^b^ Fat mass—DEXA
Kanat et al.^53^	2013	69	RCT	8	Any malignancy	Megestrol acetate + meloxicam or megestrol acetate + EPA‐enriched nutritional supplement (**pharmacological**)	Meloxicam + EPA‐enriched nutritional supplement	Body weight^b^ (*primary*) Lean body mass—BIA (*primary*) BMI
Poulsen et al.^54^	2013	61	RCT	5	Oesophageal/gastric/gynaecological	Nutritional counselling (**nutritional**)	Standard care	**Body weight** (loss vs. maintenance) (*primary*) Fat mass—BIA Fat‐free mass—BIA
Bourdel‐Marchasson et al.^55^	2014	336	RCT	10	Other mixed	Nutritional counselling (**nutritional**)	Standard care	Body weight^b^
Pottel et al.^56^	2014	85	Exploratory RCT	8	Head and neck	Echium oil (**nutritional**)	Sunflower oil	Body weight^c^ (*primary*) Lean body mass—DEXA Lean body mass—BIA Fat mass—DEXA Fat mass—BIA Fat‐free mass—BIA
Focan et al.^57^	2015	53	RCT	7	Any malignancy	Dietetic and psychological mindfulness workshops (**multi‐modal**)	Standard care	**BMI** (*primary*) **Body weight** ^b^
Garcia et al.^58^	2015	82	Phase II RCT	7	Any malignancy	Anamorelin 50 mg (**pharmacological**)	Placebo	**Lean body mass—DEXA** (*primary*) **Appendicular LBM– DEXA** **Total body mass—DEXA** Fat mass—DEXA
Capozzi et al.^59^	2016	60	Exploratory RCT	8	Head and neck	Early ‘lifestyle intervention’ (individualized exercise with education and support) (**exercise**)	Delayed ‘lifestyle intervention’ (individualized exercise with education and support)	Lean body mass—DEXA Fat mass—DEXA BMI
Kapoor et al.^60^	2016	63	RCT	8	Any malignancy	Improved atta (nutritional supplement) + nutritional counselling (**nutritional**)	Nutritional counselling	Body weight^b^ Mid‐arm circumference Various skinfold thickness
Mehrzad et al.^61^	2016	70	RCT	8	Any malignancy (not brain)	Pentoxifylline (**pharmacological**)	Placebo	Body weight^b^ Mid‐arm circumference
Stewart Coats et al.^62^	2016	87	Phase II RCT	10	NSCLC/colorectal	Espindolol 10 mg or espindolol 2.5 mg (**pharmacological**)	Placebo	**Body weight** ^b^ ^,^ ^c^ (*primary*) **Lean body mass—DEXA** Fat mass—DEXA
Takayama et al.^63^	2016	181	Phase II RCT	8	NSCLC	Anamorelin 100 mg or anamorelin 50 mg (**pharmacological**)	Placebo	**Lean body mass—DEXA** (*primary*) Lean body mass—BIA **Fat mass—DEXA** **Fat mass—BIA** **Body weight** ^b^
Temel et al.^11^	2016	979	Phase III RCT	8	NSCLC	Anamorelin (**pharmacological**)	Placebo	**Lean body mass—DEXA** (*primary*) **Body weight** ^b^ **Total body mass—DEXA** **Fat mass—DEXA** **Appendicular LBM—DEXA**
Woo et al.^64^	2016	67	Phase II RCT	9	Pancreatic	Pancreatic exocrine replacement therapy (**nutritional**)	Placebo	Body weight^b^ ^,^ ^c^ (*primary*)
Currow et al.^65^	2017	513	Phase III RCT	8	NSCLC	Anamorelin (**pharmacological**)	Placebo	**Body weight** ^b^
Jatoi et al.^66^	2017	302	RCT	8	Any malignancy (not primary brain tumour)	Creatine monohydrate (**nutritional**)	Placebo	Body weight^c^ (*primary*)
Leedo et al.^67^	2017	40	RCT	8	Lung	Home meal delivery (**nutritional**)	Standard care	Body weight^b^
Sandmael et al.^68^	2017	41	Pilot RCT	9	Head and neck	Exercise and nutrition intervention during radiotherapy treatment (**multi‐modal**)	Exercise and nutrition intervention after radiotherapy treatment	Skeletal muscle index—CT Body weight^b^
Solheim et al.^69^	2017	46	Phase II RCT	8	NSCLC/pancreatic	Exercise, celecoxib + nutritional supplements (**multi‐modal**)	Standard care	**Body weight** ^b^ ^,^ ^c^ Skeletal muscle area—CT
Werner et al.^70^	2017	60	RCT	7	Pancreatic	Fish oil (**nutritional**)	Marine phospholipids	Body weight^c^ BMI
Ziętarska et al.^71^	2017	95	RCT	6	Colorectal	Nutritional supplements (**nutritional**)	Standard care	BMI
Golan et al.^72^	2018	125	Phase II RCT	7	Pancreatic	Anti‐myostatin antibody 300 mg or anti‐myostatin antibody 100 mg (**pharmacological**)	Placebo	Thigh muscle volume—CT Skeletal muscle area—CT Adipose tissue area—CT Lean body mass—DEXA Fat mass—DEXA
Katakami et al.^73^	2018	174	Phase III RCT	8	NSCLC	Anamorelin (**pharmacological**)	Placebo	**Lean body mass—DEXA** (*primary*) **Body weight** ^b^
Kouchaki et al.^74^	2018	90	Phase III RCT	8	Gastrointestinal	Megestrol acetate + celecoxib (**pharmacological**)	Megestrol acetate + placebo	Body weight^b^ ^,^ ^c^ (*primary*)
Schink et al.^75^	2018	131	Pilot non‐randomized trial	9	Any solid tumour	Whole‐body electromyostimulation + nutritional counselling (**multi‐modal**)	Nutritional counselling	**Lean body mass—BIA** (*primary*) Fat mass—BIA **Body weight** ^b^
Uster et al.^76^	2018	58	RCT	9	Gastrointestinal/lung	Exercise programme + nutritional counselling (**multi‐modal**)	Standard care	Body weight^b^
Xie et al.^77^	2018	54	RCT	8	Lung	Thalidomide + cinobufagin (**pharmacological**)	Cinobufagin	Body weight^b^ Mid‐arm circumference
Akita et al.^78^	2019	62	RCT	8	Pancreatic	EPA‐enriched nutritional supplement (**nutritional**)	Standard care	Lean body mass—BIA Fat mass—BIA Psoas muscle area—CT BMI
Britton et al.^79^	2019	307	RCT	7	Head and neck	Psychological nutritional intervention (**nutritional**)	Standard care	**Body weight** ^c^
Cereda et al.^80^	2019	166	RCT	8	Other mixed	Whey protein isolate supplement + nutritional counselling (**nutritional**)	Nutritional counselling	**Fat‐free mass index—BIA** **Body weight** ^b^
Laviano et al.^81^	2019	55	Pilot RCT	8	NSCLC	Targeted medical nutrition supplement (**nutritional**)	Isocaloric comparator drink	Skeletal muscle area—CT Visceral fat area—CT Appendicular LBM—DEXA Fat mass—DEXA Body weight^b^
Obling et al.^82^	2019	47	RCT	7	Gastrointestinal	Supplemental home parenteral nutrition and nutritional counselling (**nutritional**)	Nutritional counselling	**Fat‐free mass—BIA** (*primary*) **Fat‐free mass index—BIA** (*primary*)
Stuecher et al.^83^	2019	44	RCT	8	Gastrointestinal	Walking exercise programme (**exercise**)	Standard care	**Lean body mass—BIA**
Wiskemann et al.^84^	2019	65	RCT	5	Pancreatic	Supervised resistance training or home‐based resistance training (**exercise**)	Standard care	Body weight^c^ (*primary*)
Boulec et al.^85^	2020	111	RCT	7	Any malignancy	Parenteral nutrition (**nutritional**)	Oral feeding	Body weight^b^
Huang et al.^86^	2020	119	RCT	7	Nasopharyngeal	Nutritional supplements (**nutritional**)	Standard care	Body weight^b^
Kamel et al.^87^	2020	40	RCT	7	Pancreatic	Resistance training (**exercise**)	Standard care	**Appendicular LBM—DEXA** **Fat mass—DEXA**
Movahed et al.^88^	2020	100	RCT	8	Oesophageal	Supplements ± enteral or parenteral nutrition ± pharmacotherapy + nutritional counselling (**multi‐modal**)	Nutritional counselling	Fat mass—BIA Fat‐free mass index—BIA Body weight^b^ BMI
Qiu et al.^89^	2020	96	RCT	6	Oesophageal	Nutritional counselling (**nutritional**)	Standard care	BMI
Storck et al.^90^	2020	52	RCT	10	Other mixed	Protein supplement + nutritional counselling + exercise programme (**multi‐modal**)	Standard care	Lean body mass—BIA Fat mass—BIA BMI
Currow et al.^91^	2021	190	Phase III RCT	6	Any malignancy	Megestrol acetate or dexamethasone (**pharmacological**)	Placebo	Body weight^b^
Hunter et al.^92^	2021	120	Phase III RCT	7	Any solid tumour	Mirtazapine (**pharmacological**)	Placebo	Lean body mass—BIA Body weight^b^
Kutz et al.^93^	2021	58	RCT	7	Head and neck	Nutritional counselling (**nutritional**)	Standard care	BMI Fat‐free mass—BIA
Tobberup et al.^94^	2021	120	Non‐randomized trial	9	NSCLC	Fish oil + nutritional counselling + exercise programme (**multi‐modal**)	Standard care (historical comparator)	Skeletal muscle area—CT Body weight^b^

*Note*: Sample sizes are reported as per ‘intention to treat’. Abbreviations: BIA, bioimpedance analysis; BMI, body mass index; CT, computed tomography; DEXA, dual‐energy X‐ray absorptiometry; EPA, eicosapentaenoic acid; LBM, lean body mass; NSCLC, non‐small‐cell lung cancer; PEG, percutaneous endoscopic gastrostomy; RCT, randomized controlled trial.

^a^ Endpoints that are bold underlined had a statistically significant difference between groups.

^b^ Endpoint expressed as change in absolute value from baseline.

^c^ Endpoint expressed as percentage change from baseline.

**Figure 2:**
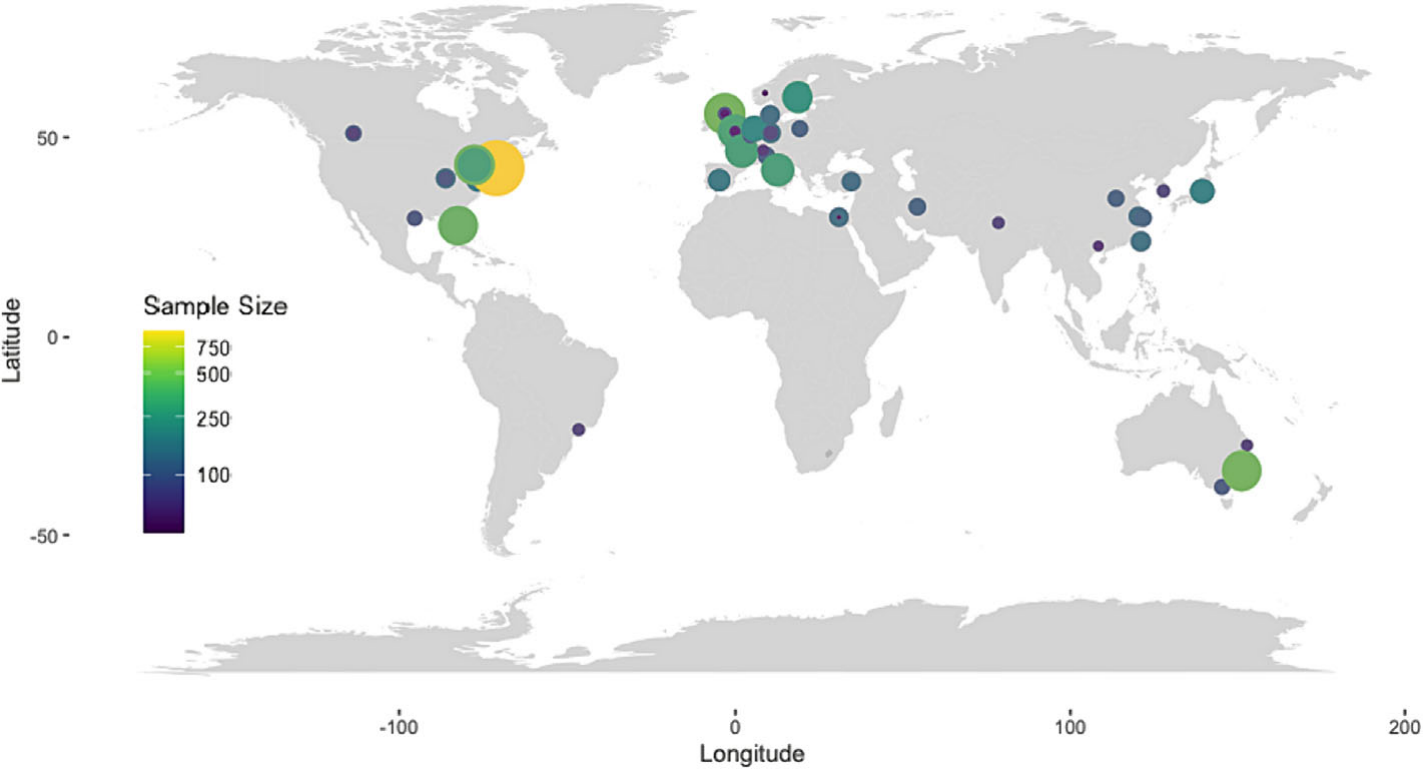
Geographical distribution of included cancer cachexia trials.

*Figure* *2* depicts the geographical distribution of the included cancer cachexia clinical trials. For multicentre or multinational trials (*n* = 12 studies), coordinates for the institution of the corresponding author were used. It was noted that limited research has been conducted in Eastern Europe, Africa or South America.

### Temporal trends in body composition endpoint selection

**Figure 3:**
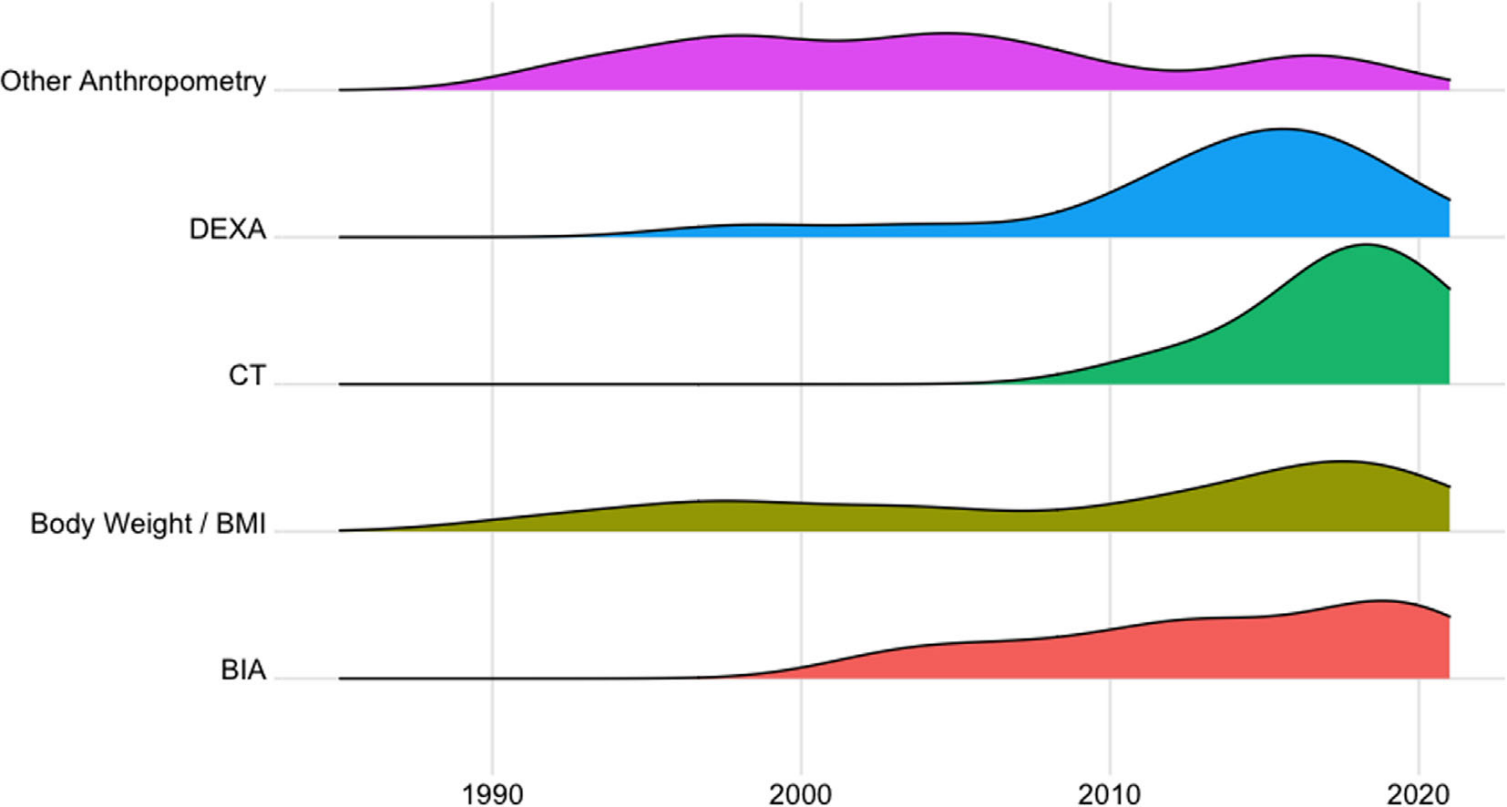
Temporal trends in relative use of body weight and body composition assessments. BIA, bioimpedance analysis; BMI, body mass index; CT, computed tomography; DEXA, dual‐energy X‐ray absorptiometry.

The relative use of body weight and body composition assessments over time is depicted in *Figure* *3*. The proportion of trials that included assessments of body weight or body mass index (BMI) as an endpoint measure did not vary particularly over the time frame considered. Other anthropometric measures (e.g., skinfold thickness or arm circumference) have been less utilized in recent years with only three trials in the last decade reporting these endpoints. BIA has been used with relative consistency during the last 20 years, whereas DEXA‐based estimates of body composition were included in only two trials before 2010, when its use increased. The reporting of CT body composition analysis in clinical trials is more contemporary with only eight trials, all conducted within the last decade, having included this assessment modality.

### Body weight and other anthropometric endpoints

**Figure 4:**
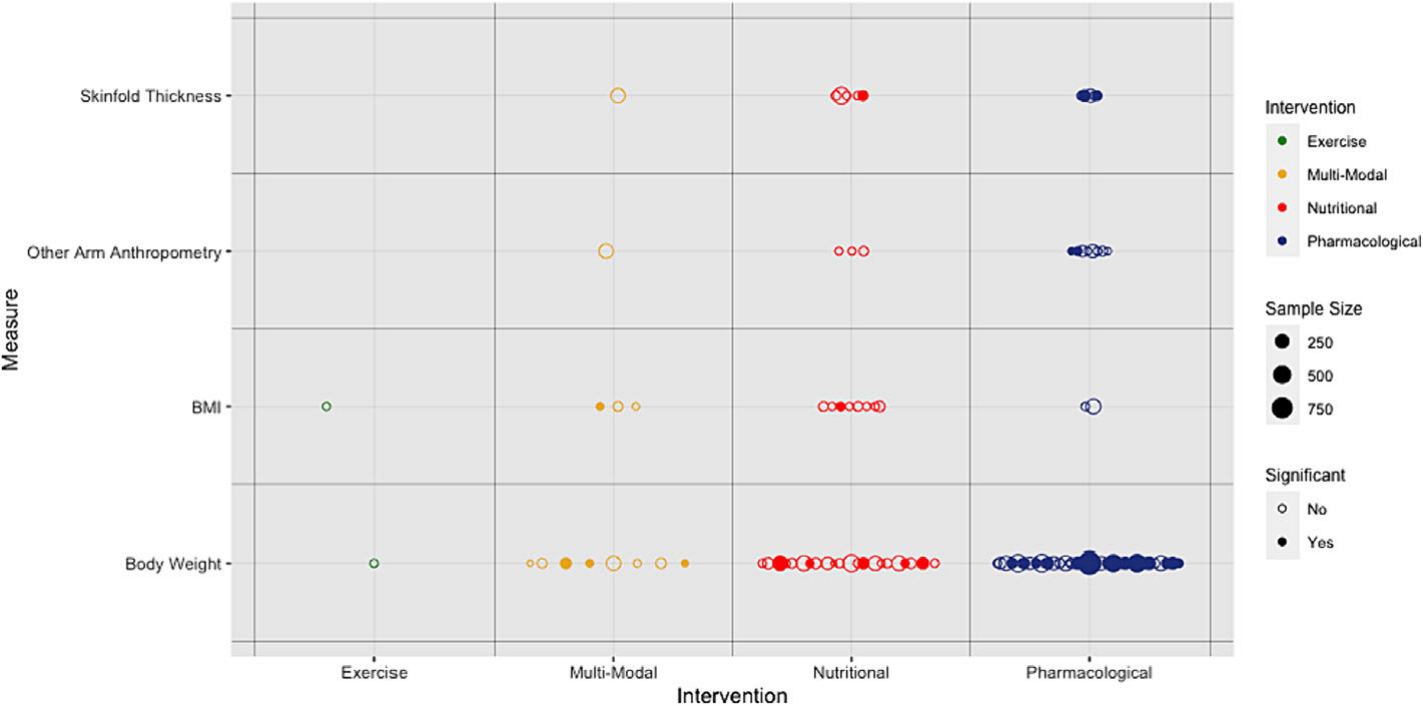
Summary of anthropometric measures of body composition by intervention modality. BMI, body mass index.

Seventy‐five trials (*n* = 12 056 participants) measured body weight or other anthropometric endpoints pertaining to body composition (*Table* *1* and *Appendix* *B*). Assessments of body weight (68 trials, *n* = 11 561 participants) were utilized in pharmacological (*n* = 37), nutritional (*n* = 22), multi‐modal (*n* = 8) and exercise‐based (*n* = 1) clinical trials. A body weight assessment was selected as the (co‐)primary endpoint in 32 (47.1%) of these trials and was a secondary/exploratory outcome for the other 36 (52.9%). Analyses were based on absolute change in body weight in 48 trials (70.6%) and percentage change from baseline in 16 trials (23.5%) with 4 trials considering both (5.9%). The ACT‐ONE trial analysed the rate (slope) of absolute and percentage weight change.^62^ Body weight was handled as an ordinal variable by three trials^19^, ^32^, ^54^ where comparison was drawn between proportions of weight‐gaining, weight‐stable and weight‐losing participants. Over one third of the trials that considered body weight (38.2%, *n* = 4735) noted significant differences between trial groups (*Figure* *4* and *Table* *2*). BMI was reported as an endpoint for 13 trials, most commonly in addition to body weight (6/13 trials). The majority of studies that chose BMI as an endpoint employed a nutritional intervention (61.5%). It was the primary endpoint in only one of these trials (7.7%). Fourteen trials included anthropometric measures of the arm as endpoints (*n* = 1901 participants). Eight of these evaluated pharmacological interventions (57.1%), five were nutritional (35.7%) and one was multi‐modal (7.1%). Measurements of mid‐arm circumference, and other derived upper arm measures such as muscle and fat areas, were selected as endpoints in 12 trials (*n* = 1324, *Appendix* *B*). Skinfold thickness was also used commonly as an endpoint (11 trials, *n* = 1727 participants). All of these 11 trials measured the triceps skinfold, and some also considered skinfold thickness at the biceps, subscapular skinfold and other sites. Triceps skinfold thickness was the primary endpoint for one trial, where arm anthropometry was used as a secondary/exploratory outcome for all other trials.

**Table 2:** Utilization of body composition endpoints

Endpoint	No. of studies	Years of publication	Total sample size	Intervention type	Statistically significant results (between trial groups)	Intervention type
Anthropometric measures						
Body weight	68	1990–2021	11 561	Pharmacological: 37 Nutritional: 22 Exercise: 1 Multi‐modal: 8	Yes: 26 No: 42	Pharmacological: 18 Nutritional: 5 Exercise/lifestyle: Multi‐modal: 3
Skinfold thickness	11	1993–2016	1727	Pharmacological: 5 Nutritional: 5 Multi‐modal: 1	Yes: 3 No: 8	Pharmacological: 2 Nutritional: 1
Other arm anthropometry	12	1993–2018	1321	Pharmacological: 8 Nutritional: 3 Multi‐modal: 1	Yes: 2 No: 10	Pharmacological: 2
Body mass index (BMI)	13	2005–2021	917	Pharmacological: 1 Nutritional: 8 Exercise: 1 Multi‐modal: 3	Yes: 2 No: 11	Nutritional: 1 Multi‐modal: 1
BIA body composition						
Lean body mass	16	2003–2021	2576	Pharmacological: 8 Nutritional: 5 Exercise: 1 Multi‐modal: 2	Yes: 2 No: 14	Exercise: 1 Multi‐modal: 1
Fat mass	8	2012–2020	744	Pharmacological: 1 Nutritional: 4 Multi‐modal: 3	Yes: 2 No: 5	Pharmacological: 1 Nutritional: 1
Fat‐free mass	6	2004–2021	384	Pharmacological: 1 Nutritional: 5	Yes: 1 No: 5	Nutritional: 1
Fat‐free mass index	3	2019–2020	313	Nutritional: 2 Multi‐modal: 1	Yes: 2 No: 1	Nutritional: 2
DEXA body composition						
Lean body mass	14	1998–2018	2957	Pharmacological: 11 Nutritional: 1 Exercise: 1 Multi‐modal: 1	Yes: 8 No: 6	Pharmacological: 8
Fat mass	11	2004–2020	2162	Pharmacological: 6 Nutritional: 2 Exercise: 2 Multi‐modal: 1	Yes: 2 No: 9	Pharmacological: 2
Appendicular lean body mass	4	2015–2020	1156	Pharmacological: 2 Nutritional: 1 Exercise: 1	Yes: 3 No: 1	Pharmacological: 2 Exercise/lifestyle: 1
Total body mass	2	2015–2016	1061	Pharmacological: 2	Yes: 2	Pharmacological: 2
CT body composition						
Estimated lean body mass (L3)	2	2010–2012	392	Pharmacological: 2	Yes: 1 No: 1	Pharmacological: 1
Skeletal muscle area (L3/L4/L5)	4	2017–2021	346	Pharmacological: 1 Nutritional: 1 Multi‐modal: 2	No: 4	N/A
Thigh muscle volume	1	2018	125	Pharmacological: 1	No: 1	N/A
Fat area (L4/L5)	1	2018	125	Pharmacological: 1	No: 1	N/A
Skeletal muscle index	1	2017	41	Multi‐modal: 1	No: 2	N/A
Psoas muscle area (L3)	1	2019	62	Nutritional: 1	No: 1	N/A
Visceral fat area	1	2019	55	Nutritional: 1	No: 1	N/A

*Note*: Sample sizes are reported as per ‘intention to treat’. Abbreviations: BIA, bioimpedance analysis; BMI, body mass index; CT, computed tomography; DEXA, dual‐energy X‐ray absorptiometry; L3/L4/L5, third/fourth/fifth lumbar vertebral level.

All studies that included arm‐based anthropometric measurements also included body weight as an endpoint. Of these, 9 (64.3%) identified no statistically significant difference between groups using any selected outcome measure. Two trials^17^, ^42^ identified statistically significant improvements in triceps skinfold thickness but no corresponding change in body weight. Conversely, McMillan et al.'s trial led to increased body weight (5.1 kg median difference between trial arms) and mid‐arm circumference measurement (1 cm median difference between trial arms), but no change in skinfold thickness measurements.^28^

### Bioelectrical body composition endpoints

Endpoints based on assessment with BIA were used in 23 trials (*n* = 3140 participants, *Table* *1* and *Appendix* *C*). The interventions tested were commonly nutritional (11 trials, 47.8%) or pharmacological (9 trials, 39.1%). BIA‐based endpoints were selected as a (co‐)primary endpoint in 6 trials (26.1%) and a secondary/exploratory endpoint in the remaining 17 (73.9%). The most frequently included endpoint was estimated whole‐body lean body mass (LBM) (16 trials, *n* = 2576 participants), with fat‐free mass (FFM) or fat‐free mass index (FFMI) calculated as alternative endpoints by 8 trials (*n* = 650 participants). Fat mass (FM) was estimated using BIA in eight trials (*n* = 744 participants).

**Figure 5:**
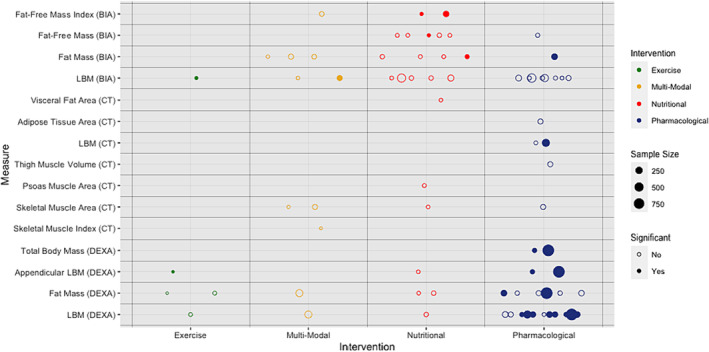
Summary of bioelectrical/radiological measures of body composition by intervention modality. BIA, bioimpedance analysis; CT, computed tomography; DEXA, dual‐energy X‐ray absorptiometry; LBM, lean body mass.

Fourteen studies (60.9%) that used BIA did not detect statistically significant differences between trial groups with any of their selected endpoints (including those not BIA‐based) (*Table* *2* and *Figure* *5*). In two trials, improvements were identified in BIA estimates of LBM/FFM that were congruent with increased body weight.^75^, ^80^ A statistically significant increase in body weight was identified in two trials of nutritional counselling^35^, ^54^ but there were no accompanying changes in BIA estimates of LBM. Two of the seven trials (28.6%) that estimated FM using BIA demonstrated a statistically significant increase with their intervention that was congruent with body weight gain.^46^, ^63^

### Radiological body composition endpoints

**Figure 6:**
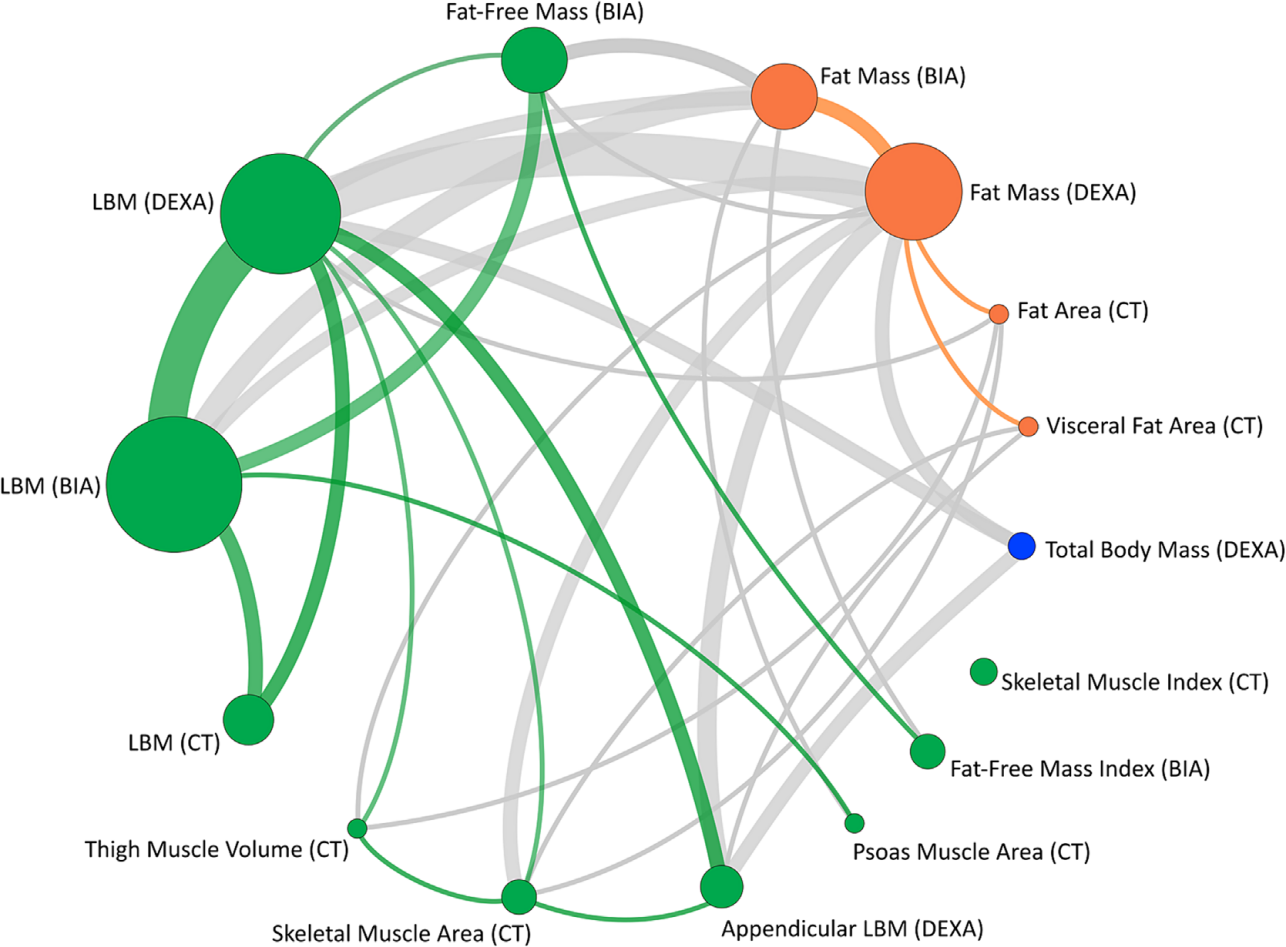
A network diagram illustrating combinations of reported bioelectrical/radiological endpoints. Endpoints pertaining to muscle are highlighted in green. Endpoints pertaining to fat are highlighted in orange. Green connecting lines highlight the use of two measures of muscle in the same trial. Similarly, orange lines highlight the use of two measures of fat, while grey lines indicate combinations of muscle and fat measures. The size of the nodes reflects the number of studies that have reported the endpoint. The thickness of the connecting line reflects the number of studies reporting each pair of measures. BIA, bioimpedance analysis; CT, computed tomography; DEXA, dual‐energy X‐ray absorptiometry; LBM, lean body mass.

DEXA was used in 16 trials (*n* = 3052) with LBM (*n* = 2957 participants) and FM (*n* = 2162) being the most frequently reported endpoints (*Table* *1* and *Appendix* *C*). Appendicular lean mass was used as an alternative endpoint in two trials^81^, ^87^ and alongside whole‐body LBM in another two trials^58^, ^65^ (*Figure* *6*). Pharmacological interventions were used for most trials that used DEXA (68.8%). DEXA‐based measures were used as the primary endpoint for 50% (*n* = 8) of these trials.

Five trials included in this review compared the effects of anamorelin against placebo (*n* = 1929 participants). Of these, four evaluated LBM using DEXA, and all identified a statistically significant increase compared to placebo with congruent increases in overall body weight.^11^, ^58^, ^63^, ^73^ Of note, Takayama et al.'s relatively large (*n* = 181) placebo‐controlled trial identified significant improvements in body weight alongside increased FM using both DEXA and BIA, but only a significant improvement in LBM when measured with DEXA (mean difference vs. placebo: 1.15 kg [95% confidence interval—CI: 0.11–2.18]), not with BIA‐based estimates (mean difference vs. placebo: 0.78 kg [95% CI: −0.35 to 1.90]).^63^

Only eight trials included endpoints based on CT body composition (*n* = 841, *Table* *1* and *Appendix* *C*), with two of these (25%) considering it a (co‐)primary endpoint. All but one^43^ of these had relatively small sample sizes (≤125 patients). Four measured the cross‐sectional area of skeletal muscle (*n* = 346) at the third (L3)^68^, ^69^, ^94^ or fourth/fifth lumbar vertebral level.^72^ One of these studies reported the L3 cross‐sectional area of muscle as normalized for height, termed skeletal muscle index (SMI).^68^ Others included the L3 cross‐sectional area of psoas major^78^ or derived estimates of LBM (kg) based on L3 muscularity.^43^, ^48^ CT estimates of adipose tissue were also reported by two trials.^72^, ^81^ Six of the included trials noted no significant differences for any of their selected endpoints.

The five‐arm phase III randomized controlled trial (RCT) (*n* = 332) by Mantovani et al.^43^ identified improved LBM using DEXA (mean difference: 2.1 kg) and CT estimates (mean difference: 2.6 kg) in one of the trial arms but detected no difference using BIA (mean difference: 1.2 kg, *P* = 0.609). Similarly, Madeddu et al.^48^ found improvements in LBM across both trial arms based on DEXA and CT estimates, but not with BIA.

## Discussion

This systematic review summarizes the frequency and diversity of endpoints examining body weight and composition in cancer cachexia clinical trials. It is one of six systematic reviews being undertaken, with others considering physical function,^9^ quality of life, appetite and dietary intake, biomarkers and oncology/survival endpoints. Assessments of body weight were the most commonly reported endpoint, used by over 80% of the included trials. Other anthropometric measures, such as skinfold thickness measurements and arm circumference, were less frequently used, especially in more contemporary trials. BIA‐based estimates were often included but have been largely superseded by DEXA, especially in larger trials, and more recently by CT body composition analyses.

Body weight is the simplest and most widely available assessment that can indicate alterations to body composition and has long been regarded as a central tenet of cachexia. Included trials have used it to create various endpoints (e.g., absolute/percentage differences from baseline or comparison between weight‐stable, weight‐gaining and weight‐losing groups). Further study is likely required to establish consensus on which of these specific body weight endpoints is most informative. While BMI is likely a helpful baseline descriptor, given the relevance of obesity as a prognostic variable in patients with cachexia and/or sarcopenia,^95^, ^96^, ^97^ the use of BMI as an endpoint instead of, or alongside, body weight does not add value. Other simple anthropometric measures, such as triceps skinfold thickness or other arm anthropometry, were featured in earlier cachexia trials but have been used less in recent years. All of these methods pose practical advantages, such as low cost, widespread availability and ease of measurement, but provide limited information when compared with radiological methods.

Estimates of body composition using BIA have been featured as chosen endpoints for a number of the included trials. BIA measures the electrical properties of tissues (resistance and reactance), and these values can be used in equations to approximate FM/FFM/LBM. Previous studies have shown that BIA is prone to overestimation for lean mass and underestimation of FM^98^ and has poor agreement with comparable DEXA^99^ and CT‐based measures.^100^ Indeed, some of the included studies identified significant differences between trial groups on DEXA and/or CT estimates of body composition that were not evident using BIA‐based estimates.^43^, ^48^, ^63^ However, it must be noted that BIA does have several practical advantages. It uses equipment that is portable and non‐invasive, making it perhaps a more attractive option for assessments in frail, incurable cachexia populations or in community‐based studies where assessments may take place in non‐clinical locations. As such, BIA may remain an appropriate option for particular trial settings despite its limitations.

DEXA has been widely used for assessment of lean and adipose tissue in cachexia trials to date, including amongst many of the larger studies included in this review.^11^, ^36^, ^43^ It does not routinely feature during the clinical staging pathway for patients with cancer but is an attractive research imaging adjunct owing to its modest cost, low radiation dose and short imaging time. While DEXA estimates of LBM have shown excellent correlation with both MRI and CT,^101^ there is some evidence that measures of adipose tissue using DEXA can tend towards underestimation.^102^ It also lacks the specificity to assess changes in individual muscle groups and is unable to identify myosteatosis, which appears to be an important prognostic feature on CT^103^ and MRI body composition analyses^6^ via observational studies.

CT body composition analysis was used in eight contemporary cancer cachexia trials (2010 onwards). Within the broader literature, a large number of observational studies have evaluated the impact of low muscle quantity^104^ or radiodensity^103^ as per CT body composition analysis, with consistently adverse prognostication noted for patients with cancer. While only one of the included trials identified significant differences between groups using CT‐based endpoints, it must be noted that similar negative findings were seen on other concurrent endpoints (including body weight, DEXA and BIA) in all but one study.^69^ This is therefore likely reflective of the efficacy of the interventions used, rather than the sensitivity of the endpoint measured. Furthermore, some of the trials that included CT‐based assessments used muscle groups or vertebral levels that have not yet been adequately validated to the same standard as the use of L3 cross‐sectional area.^3^ Other assessments, such as radiodensity or volumetric body composition analysis, remain unexplored in the cachexia trial setting. CT is a very pragmatic imaging modality for some patient groups, owing to its routine use in the clinical staging and follow‐up of cancer patients. However, when research imaging requirements are in excess of clinical need, the issue of associated ionizing radiation must be duly considered. Furthermore, trial timepoints may not align with the clinical pathway. Balancing this alongside finite resources and the need to limit patient burden can be challenging. Despite the dose reductions achieved with improved technology and focused scanning,^105^ the necessary radiation exposure from CT still often exceeds that of DEXA.^106^ As such, when clinical imaging is not available, other assessment modalities such as MRI may be more appropriate.

Non‐significant findings may result from an intervention that lacks efficacy, a trial that is inadequately powered relative to the chosen outcome or a measurement that lacks the precision to detect a true effect. With this in mind, limited inferences may be drawn regarding how a trial's significant or non‐significant results may reflect on the selected endpoint(s), particularly if these are secondary endpoints that were not featured during sample size calculations. When comparing methods of body composition assessment, it is useful to consider several key measurement characteristics: reliability, validity, responsiveness to change, minimally important clinical difference and sensibility.

Reliability refers to the consistency and repeatability of a measurement when applied under similar conditions. One could consider intra‐rater reliability (agreement of measures by a single evaluator on different occasions) or inter‐rater reliability (agreement between different evaluators). Body weight would be expected to have excellent inter‐rater reliability, owing to ease of measurement. However, achieving intra‐rater reliability is dependent on consistent patient factors (e.g., clothing or fasting and hydration status) and use of the calibrated instruments. While good intra‐rater and inter‐rater reliability has been demonstrated for BIA, this also requires adherence to strict standardization of measurement conditions,^107^ which may be difficult to achieve in real‐world settings. DEXA and CT assessments of body composition may previously have been subject to lower levels of reliability, owing to their need for manual segmentation of anatomical features or regions of interest, but technological and software advancements have led to more reliable measurements now being obtained.^108^ Furthermore, CT performs well during tests of precision; the ability of a measurement technique to reproduce results when performed in an identical manner.^109^

Validity refers to a method's accuracy in assessing what it is intended to measure. In the case of cachexia, researchers are likely to be interested in changes in quantities of muscle and/or fat. This may present an obvious limitation with body weight, as it cannot inform us regarding the alterations to body composition that have led to any change in weight. This limits its use in isolation. Furthermore, the potential for fluid accumulations (e.g., ascites/peripheral oedema/hydration status) to influence body weight, BIA or even DEXA could lead to these modalities providing less valid assessments.^110^ While most studies of CT body composition extrapolate single‐slice measurements to estimate whole‐body composition, it is not known whether wasting occurs uniformly throughout the body.^109^ Analyses conducted over larger regions of interest may yet improve the validity of CT body composition.^111^

Responsiveness to change is a measure's ability to detect meaningful differences over time and is crucial for monitoring responses to trial interventions. Multiple factors can influence these parameters, and in the setting of cachexia clinical trials, it can be challenging to assess efficacy independent of confounders. While single axial slice CT (e.g., L3) and whole‐body measurements are known to be highly correlated,^3^ this may not hold true when assessing changes over time.^109^

A minimal clinically important difference (MCID) describes the smallest change that could be considered clinically significant. Such a metric may be determined with consideration of how changes in body composition endpoints relate to other outcomes. For example, what change in muscle mass is required to influence quality of life or survival? As has been observed with muscle mass and function,^112^ the relationships between endpoints may be non‐linear, and this must be acknowledged when considering responsiveness to change. The minimum body weight change that is considered clinically important for an individual's health is more commonly studied in the field of obesity than in cachexia. Semaglutide (glucagon‐like peptide‐1 [GLP‐1] receptor agonist) was granted Food and Drug Administration (FDA) approval based on >5% weight loss being regarded as clinically meaningful.^113^ A >5% weight gain may be considered an equivalent MCID for treatment of cachexia, yet such precedent has not been set thus far.

Measurements should have sensibility (or interpretability) so they can be understood with ease. Body weight is meaningful and can be easily interpreted by clinicians, researchers and even patients. Conversely, bioelectrical estimates of body composition are less well known, and as such, the sensibility of these is limited. With improved consensus regarding effective assessment methods and endpoints, it should be anticipated that relevant stakeholders will become more informed regarding their chosen measurement techniques and how their findings relate to patients.

It is evident that no single assessment method currently fulfils all requirements. Rather, researchers should choose appropriate endpoints to align with their study aims and the cohort in question. What represents the ‘gold‐standard’ measure would be dependent on the choice of intervention and its underlying mechanism. Assessment of body weight alongside dietary intake may be reasonable when assessing an appetite stimulant and has the added advantage of having regulatory approval in the obesity arena. Similarly, assessing DEXA or CT‐based body composition would be sensible when trialling an exercise intervention aimed to improve lean mass. The practicalities, including cost and participant burden, mean that assessing these in clinical trials may be aspirational, despite a clear need.

A large volume of data has been compiled through each of the six reviews undertaken within this series. While there was a need to give a detailed appraisal within each of these, further work is ongoing to examine the relationships between these parameters. The findings presented are likely to have even greater value when considered in the context of other endpoints. For example, how do improvements in lean mass relate to physical function? The group aspires towards achieving a wider consensus alongside the identification and prioritization of areas for future research. Key strengths of this review include the broad search criteria and the robust methodological approach and appraisal process. However, the eligibility criteria could be considered a limitation, as balance was sought between the need to find trials of sufficient quality against having to appraise an impractical number of manuscripts. Although a specific time period was defined for the purposes of this work, it is accepted that trials published before 1990 may have yielded additional data, though this would pre‐date the use of most endpoints considered by this review. The sample size cut‐off was felt to be appropriate, as trials with <40 participants were expected to be insufficiently powered to assess changes in the endpoints being assessed. Furthermore, the minimum intervention time of 14 days was selected as interventions conducted for a shorter duration were felt unlikely to influence the disease course of cachexia. It should be acknowledged, however, that these restrictions may have precluded the inclusion of some informative trials. While the focus of this review was prospective, interventional trials, it is also acknowledged that many high‐quality studies of other designs may yet inform the process of establishing a consensus regarding the optimum endpoints for cachexia trials, and future review of these would also be informative.

Endpoints should be intrinsically linked to inclusion criteria and vice versa. Changes in body composition are a key feature of cancer cachexia and, therefore, by definition, often feature as a baseline descriptive criterion (and, thus, outcome measure) in cachexia intervention trials. The Global Leadership Initiative on Malnutrition (GLIM) consensus criteria^114^ were not designed to be used for the diagnosis of cachexia; however, they are intended to complement the existing cachexia literature, acknowledging that all patients with cachexia would meet their diagnosis of malnutrition. This group agreed that a body composition‐based phenotypical criterion (weight loss, low BMI or reduced muscle mass) and an aetiological criterion (reduced food intake/assimilation or inflammation) are required for a diagnosis of malnutrition. Their recommendation for methods of estimating low muscle mass was for DEXA or ‘corresponding standards using other body composition methods like BIA, CT or MRI’. The group also stated that anthropometric measures, such as arm muscle circumference, may be used as an alternative when radiological imaging is unavailable. Similarly, broad assessment methodology was proposed by the European consensus definition for sarcopenia,^5^ with DEXA, BIA, CT and MRI all listed as options for evaluating ‘muscle quantity or quality’. As shown in this present review, the range of modalities within the guidance is reflective of the heterogeneity within the existing literature.

## Conclusions

Based on the findings presented herein, the use of body weight alongside a radiological modality for body composition analysis would seem like suitable endpoints for cancer cachexia trials. Thus far, body weight has been reported in a variety of ways, and further consensus is required regarding the specific body weight endpoint that should be used for future trials. The choice of radiological modality is likely to be dependent on the trial setting, population and intervention in question. When available, CT imaging is a well‐validated and often pragmatic option that provides good levels of detail regarding body composition. Through ongoing exploration of this, and other assessment methods such as MRI,^110^ further evidence is likely to emerge that will help standardize the appraisal of body composition. Endpoint heterogeneity in cancer cachexia clinical trials has greatly contributed to the lack of approved treatments by regulatory authorities.^115^ Moreover, discrepancies between clinicians, regulatory industries and patients' perspectives regarding the most clinically relevant endpoints in cancer cachexia remain challenging. It is vital that consensus is achieved to ensure reporting consistency and maximize the efficacy of upcoming trials aiming to counteract the devastating effects of cancer cachexia.

## Conflict of interest statement

LRB, MSS, MSY, VEB, DCM, AB, TRB, OD, RDD, MTF, CG, MJH, GJ, MM, JM, IOO, IP, JS, MRS, OMV and TSS have none to declare. JA has received lecture fees from Baxter and Danone. RJES has received personal fees for consultancy from Avidity Biosciences, Actimed, Faraday and Helsinn. BJAL has received personal fees for consultancy from Artelo, Actimed, Faraday, Kyowa Kirin and Toray.

## Appendix A Literature search strategy

1. Documentation on the literature search for ‘What is the optimal endpoint to evaluate effect of interventions aiming to treat cancer cachexia?’

The following databases were searched:

**Table:**

Database	Number of retrieved references for trials	Number of retrieved references for cohort/longitudinal studies
MEDLINE (Ovid)	3812	1918
Embase (Ovid)	2033	2031
Cochrane Central Register of Controlled Trials	1923	
Number of references before de‐duplication	8166	3949
Number of references after de‐duplication	5998	3190

All searches were done on 2 June 2021 by Gunn Kleven, senior librarian at the Library of Medicine and Science, University of Oslo.

Number of hours spent: 30

**Ovid MEDLINE** ^**®**^ **ALL** 1946 to 1 July 2021

Date searched: 2 June 2021

Search strategy:

**Table:**

#	Searches	Results
1	exp Neoplasms/or (neoplasm* or cancer* or tumor* or tumour* or oncol* or malign* or carcinom* or adenocarcinom* or adenoma or metasta*).ti,ab,kf.	4 606 655
2	Cachexia/ or Emaciation/ or Malnutrition/ or Starvation/ or Wasting syndrome/ or Thinness/ or Sarcopenia/ or Anorexia/or *Weight Loss/	63 432
3	and/1‐2	9650
4	((cachexia or cachexic or anorexia or anorectic or emaciat* or malnutrition or underweight or starvation* or thiness or leanness or sarcopenia or wasting syndrome* or wasting disease* or weightloss* or ((appetite* or weight) adj2 (loss or loosing or losing))) adj4 (neoplasm* or cancer* or tumor* or tumour* or oncol* or malign* or carcinom* or adenocarcinom* or adenoma or metasta*)).ti,ab,kf.	7231
5	((cachexia or cachexic or anorexia or anorectic or emaciat* or malnutrition or underweight or starvation* or thiness or leanness or sarcopenia or wasting syndrome* or wasting disease* or weightloss* or ((appetite* or weight) adj2 (loss or loosing or losing))) and (neoplasm* or cancer* or tumor* or tumour* or oncol* or malign* or carcinom* or adenocarcinom* or adenoma or metasta*)).ti.	4253
6	or/3‐5	13 924
7	randomized controlled trial.pt.	536 354
8	controlled clinical trial.pt.	94 265
9	randomized.ab.	525 221
10	placebo.ab.	219 320
11	drug therapy.fs.	2 343 029
12	randomly.ab.	360 557
13	trial.ab.	557 904
14	groups.ab.	2 213 680
15	or/7‐14	5 047 938
16	exp animals/not humans.sh.	4 855 037
17	15 not 16	4 388 865
18	6 and 17	4078
19	limit 18 to yr = ‘1990‐Current’	3812
20	cohort studies/ or follow‐up studies/ or longitudinal studies/ or ‘national longitudinal study of adolescent health’/ or prospective studies/or retrospective studies/	2 168 707
21	(cohort* or longitudinal or prospective* or retrospective*).tw.	2 098 508
22	or/20‐21	3 012 965
23	and/6,22	3215
24	limit 23 to yr = ‘1990‐Current’	3139
25	24 not 19	1918
26	19 or 24	5730

**Embase Classic + Embase** 1947 to 1 July 2021

Date searched: 2 June 2021

Search strategy:

**Table:**

#	Searches	Results
1	exp neoplasm/or (neoplasm* or cancer* or tumor* or tumour* or oncol* or malign* or carcinom* or adenocarcinom* or adenoma or metasta*).ti,ab,kw.	6 400 926
2	cachexia/ or emaciation/ or *malnutrition/ or starvation/ or wasting syndrome/ or *anorexia/ or sarcopenia/or *weight loss/	93 608
3	and/1‐2	20 654
4	((cachexia or cachexic or anorexia or anorectic or emaciat* or malnutrition or underweight or starvation* or thiness or leanness or sarcopenia or wasting syndrome* or wasting disease* or weightloss* or ((appetite* or weight) adj2 (loss or loosing or losing))) adj3 (neoplasm* or cancer* or tumor* or tumour* or oncol* or malign* or carcinom* or adenocarcinom* or adenoma or metasta*)).ti,ab,kw.	9798
5	((cachexia or cachexic or anorexia or anorectic or emaciat* or malnutrition or underweight or starvation* or thiness or leanness or sarcopenia or wasting syndrome* or wasting disease* or weightloss* or ((appetite* or weight) adj2 (loss or loosing or losing))) and (neoplasm* or cancer* or tumor* or tumour* or oncol* or malign* or carcinom* or adenocarcinom* or adenoma or metasta*)).ti.	6437
6	or/3‐5	24 964
7	Randomized controlled trial/	666 248
8	Controlled clinical trial/	463 928
9	random$.ti,ab.	1 691 555
10	randomization/	91 413
11	intermethod comparison/	272 763
12	placebo.ti,ab.	330 754
13	(compare or compared or comparison).ti.	571 937
14	((evaluated or evaluate or evaluating or assessed or assess) and (compare or compared or comparing or comparison)).ab.	2 334 019
15	(open adj label).ti,ab.	88 467
16	((double or single or doubly or singly) adj (blind or blinded or blindly)).ti,ab.	251 401
17	double blind procedure/	187 924
18	parallel group$1.ti,ab.	27 750
19	(crossover or cross over).ti,ab.	112 863
20	((assign$ or match or matched or allocation) adj5 (alternate or group$1 or intervention$1 or patient$1 or subject$1 or participant$1)).ti,ab.	359 989
21	(assigned or allocated).ti,ab.	424 479
22	(controlled adj7 (study or design or trial)).ti,ab.	385 967
23	(volunteer or volunteers).ti,ab.	264 717
24	human experiment/	550 005
25	trial.ti.	340 818
26	or/7‐25	5 516 795
27	(random$ adj sampl$ adj7 (cross section$ or questionnaire$1 or survey$ or database$1)).ti,ab. not (comparative study/ or controlled study/or randomi?ed controlled.ti,ab. or randomly assigned.ti,ab.)	8736
28	Cross‐sectional study/not (randomized controlled trial/ or controlled clinical study/ or controlled study/or randomi?ed controlled.ti,ab. or control group$1.ti,ab.)	273 554
29	(((case adj control$) and random$) not randomi?ed controlled).ti,ab.	18 641
30	(Systematic review not (trial or study)).ti.	179 350
31	(nonrandom$ not random$).ti,ab.	17 184
32	Random field$.ti,ab.	2525
33	(random cluster adj3 sampl$).ti,ab.	1368
34	(review.ab. and review.pt.) not trial.ti.	902 488
35	we searched.ab. and (review.ti. or review.pt.)	37 332
36	update review.ab.	116
37	(databases adj4 searched).ab.	43 700
38	(rat or rats or mouse or mice or swine or porcine or murine or sheep or lambs or pigs or piglets or rabbit or rabbits or cat or cats or dog or dogs or cattle or bovine or monkey or monkeys or trout or marmoset$1).ti. and animal experiment/	1 113 420
39	Animal experiment/not (human experiment/or human/)	2 339 504
40	or/27‐39	3 735 626
41	26 not 40	4 907 360
42	and/6,41	3904
43	limit 42 to yr = ‘1990‐Current’	3674
44	limit 43 to conference abstracts	1641
45	43 not 44	2033
46	cohort analysis/ or follow up/ or longitudinal study/ or ‘national longitudinal study of adolescent health’/ or prospective study/or retrospective study/	3 499 550
47	((cohort adj (study or studies)) or cohort analy* or longitudinal).tw.	705 575
48	or/46‐47	3 722 608
49	and/6,48	4417
50	limit 49 to yr = ‘1990‐Current’	4387
51	limit 50 to conference abstracts	1639
52	50 not 51	2748
53	52 not 45	2031

**Cochrane Central Register of Controlled Trials**

Date searched: 2 June 2021

Search strategy:

**Table:**

#	Searches	Results
1	[mh Neoplasms]	82 548
2	((neoplasm* or cancer* or tumor* or tumour* or oncol* or malign* or carcinom* or adenocarcinom* or adenoma or metasta*)):ti,ab,kw (Word variations have been searched)	232 559
3	#1 or #2	241 300
4	[mh Cachexia] or [mh ^Emaciation] or [mh ^Malnutrition] or [mh Starvation] or [mh ^‘Wasting syndrome’] or [mh Thinness] or [mh Sarcopenia] or [mh Anorexia]	2665
5	MeSH descriptor: [Weight Loss] this term only	6360
6	#3 and (#4 or #5)	1017
7	(((cachexia or cachexic or anorexia or anorectic or emaciat* or malnutrition or underweight or starvation* or thiness or leanness or sarcopenia or ‘wasting syndrom’ or ‘wasting syndromes’ or ‘wasting disease’ or ‘wasting diseases’ or weightloss* or ((appetite* or weight) near/2 (loss or loosing or losing))) near/3 (neoplasm* or cancer* or tumor* or tumour* or oncol* or malign* or carcinom* or adenocarcinom* or adenoma or metasta*))):ti,ab,kw	1475
8	(((cachexia or cachexic or anorexia or anorectic or emaciat* or malnutrition or underweight or starvation* or thiness or leanness or sarcopenia or ‘wasting syndrome’ or ‘wasting syndromes’ or ‘wasting disease’ or ‘wasting diseases’ or weightloss* or ((appetite* or weight) near/2 (loss or loosing or losing))) and (neoplasm* or cancer* or tumor* or tumour* or oncol* or malign* or carcinom* or adenocarcinom* or adenoma or metasta*))):ti (Word variations have been searched)	758
9	#6 or #7 or #8 with Publication Year from 1990 to 2021, in Trials	2345

## Appendix B Included studies considering anthropometric assessments of body composition

**Table:**

Author (reference)	Year	Sample size	Study design	Study quality	Primary cancer site	Intervention	Comparator	Body composition outcomes^b^
Body weight								
Kardinal et al.^12^	1990	293	RCT	8	Any malignancy (not brain)	Cyproheptadine (pharmacological)	Placebo	Body weight^d^ (*primary*)
Loprinzi et al.^13^	1990	133	RCT	9	Any malignancy (not brain/breast/endometrial)	Megestrol acetate (pharmacological)	Placebo	**Body weight** ^c^ ^,^ ^d^ (*primary*)
Feliu et al.^14^	1992	150	RCT	5	Any malignancy (not hormone dependent)	Megestrol acetate (pharmacological)	Placebo	Body weight^c^ (*primary*)
Downer et al.^15^, ^a^	1993	60	RCT	1	Any malignancy	Medroxyprogesterone acetate (pharmacological)	Placebo	Body weight^c^ (*primary*) Mid‐arm circumference Triceps skinfold thickness
Loprinzi et al.^16^	1993	342	Phase III RCT	8	Any malignancy (not breast/endometrial)	Megestrol acetate 1280 mg or megestrol acetate 800 mg (pharmacological)	Megestrol acetate 480 mg or megestrol acetate 160 mg	Body weight^d^ (*primary*)
Ovesen et al.^17^, ^a^	1993	105	RCT	8	Small‐cell‐lung/ovarian/breast	Nutritional counselling (nutritional)	Standard care	Body weight^c^ (*primary*) Arm muscle area **Triceps skinfold thickness**
Goldberg et al.^18^	1995	70	RCT	8	Any malignancy (not primary brain tumour)	Pentoxifylline (pharmacological)	Placebo	Body weight^d^ (*primary*)
Gebbia et al.^19^	1996	122	RCT	6	Any malignancy (not hormone dependent)	Megestrol acetate 320 mg (pharmacological)	Megestrol acetate 160 mg	Body weight (*primary*)
Lissoni et al.^20^	1996	100	RCT	7	Any solid tumour	Melatonin (pharmacological)	Standard care	**Body weight** ^c^ (*primary*)
Simons et al.^21^	1996	206	RCT	7	Any malignancy (not hormone dependent)	Medroxyprogesterone acetate (pharmacological)	Placebo	**Body weight** ^c^ (*primary*)
Beller et al.^22^, ^a^	1997	240	RCT	4	Any malignancy (not hormone dependent)	Megestrol acetate 480 mg or megestrol acetate 160 mg (pharmacological)	Placebo	Body weight^c^ (*primary*) Mid‐arm circumference Mid‐arm fat area Mid‐arm muscle area Triceps skinfold thickness
Chen et al.^23^	1997	129	RCT	8	Head and neck	Megestrol acetate or prepulside (pharmacological)	Placebo	**Body weight** ^c^ (*primary*)
Daneryd et al.^24^	1998	180	RCT	7	Any malignancy	Indomethacin + erythropoietin (pharmacological)	Indomethacin	Lean body mass—DEXA **Body weight** ^c^
De Conno et al.^25^	1998	42	RCT	6	Any malignancy (not hormone dependent)	Megestrol acetate (pharmacological)	Placebo	**Body weight** ^c^ (*primary*)
Vadell et al.^26^, ^a^	1998	150	RCT	5	Any malignancy	Megestrol acetate 480 mg or megestrol acetate 160 mg (pharmacological)	Placebo	**Body weight** ^c^ (*primary*) Mid‐arm circumference **Triceps skinfold thickness**
Loprinizi et al.^27^	1999	496	RCT	8	Any malignancy (not breast/prostate/ovarian/endometrial)	Megestrol acetate or dexamethasone (pharmacological)	Fluoxymesterone	Body weight^d^ (*primary*)
McMillan et al.^28^, ^a^	1999	73	RCT	7	Gastrointestinal	Megestrol acetate + ibuprofen (pharmacological)	Megestrol acetate + placebo	**Body weight** ^c^ (*primary*) **Mid‐arm circumference** Triceps skinfold thickness Biceps skinfold thickness
Westman et al.^29^	1999	255	RCT	7	Other mixed	Megestrol acetate (pharmacological)	Placebo	Body weight^c^ (*primary*)
Jatoi et al.^30^	2002	469	RCT	10	Any malignancy (not brain/breast/ovarian/endometrial)	Megestrol acetate + dronabinol or megestrol acetate + placebo (pharmacological)	Dronabinol + placebo	**Body weight** ^d^ (*primary*)
Persson et al.^31^	2002	144	RCT	6	Breast/colorectal/gastric/prostate	Individual nutritional counselling or individual and group nutritional counselling (nutritional)	Group nutritional counselling or standard care	**Body weight** ^d^ (*primary*)
Ulutin et al.^32^	2002	119	RCT	9	NSCLC	Megestrol acetate 320 mg (pharmacological)	Megestrol acetate 160 mg	**Body weight** (increase vs. stable vs. decrease)
Bruera et al.^33^, ^a^	2003	91	RCT	7	Any malignancy	Fish oil capsules (nutritional)	Placebo	Lean body mass—BIA Body weight^c^ Mid‐arm muscle circumference Triceps skinfold thickness Subscapular skinfold thickness
Fearon et al.^34^	2003	200	RCT	8	Pancreatic	*n*‐3 fatty acid and antioxidant‐enriched supplement (nutritional)	Supplement without *n*‐3 fatty acid and antioxidants	Lean body mass—BIA Body weight^c^
Isenring et al.^35^	2004	60	RCT	8	Gastrointestinal/head and neck	Nutrition counselling and protocol (nutritional)	Standard care	Fat‐free mass—BIA **Body weight** ^c^
Lundholm et al.^36^, ^a^	2004	309	RCT	5	Any solid tumour	Indomethacin + erythropoietin + nutritional support + home total parenteral nutrition (multi‐modal)	Indomethacin + erythropoietin	Fat mass—DEXA Lean body mass—DEXA Body weight^c^ Mid‐arm muscle circumference Triceps skinfold thickness
Gonçalves Dias et al.^37^, ^a^	2005	64	Non‐randomized trial	1	Head and neck	Home enteral (nasogastric) feeding or oral diet + nutritional supplements (nutritional)	Oral diet	Body weight^c^ BMI Mid‐arm circumference Mid‐arm muscle area Triceps skinfold thickness
Gordon et al.^38^, ^a^	2005	50	RCT	10	Pancreatic	Thalidomide (pharmacological)	Placebo	**Body weight** ^c^ (*primary*) **Bone‐free arm muscle area**
Fearon et al.^39^	2006	518	RCT	8	Gastrointestinal/lung	EPA 2 g or EPA 4 g (pharmacological)	Placebo	Lean body mass—BIA Body weight^c^
Berk et al.^40^, ^a^	2008	472	RCT	9	Any solid tumour	Nutritional supplement (nutritional)	Placebo	Lean body mass—BIA (*primary*) Body weight^d^ Various skinfold thickness
Beijer et al.^42^, ^a^	2009	100	RCT	8	Any malignancy	Adenosine 5′‐triphosphate (pharmacological)	Standard care	**Triceps skinfold thickness** (*primary*) Body weight^c^ Mid‐arm circumference
Navari et al.^44^	2010	80	RCT	7	Gastrointestinal/lung	Megestrol acetate + olanzapine (pharmacological)	Megestrol acetate	**Body weight** ^d^ (*primary*)
Baldwin et al.^45^	2011	358	RCT	8	Gastrointestinal/NSCLC/mesothelioma	Nutritional supplement + nutritional counselling or nutritional supplement (nutritional)	Nutritional counselling or standard care	Body weight^c^
Silander et al.^49^, ^a^	2012	134	RCT	6	Head and neck	Prophylactic PEG (nutritional)	Standard care	Body weight^d^ (*primary*) BMI
Wen et al.^50^	2012	102	RCT	5	Any malignancy	Megestrol acetate + thalidomide (pharmacological)	Megestrol acetate	**Body weight** ^c^ (*primary*)
Del Fabbro et al.^51^	2013	73	RCT	10	Gastrointestinal/lung	Melatonin (pharmacological)	Placebo	Body weight^c^ (*primary*) Lean body mass—BIA Fat‐free mass—BIA
Dobs et al.^52^	2013	159	Phase II RCT	8	Other mixed	Enobosarm 1 mg or enobosarm 3 mg (pharmacological)	Placebo	**Lean body mass—DEXA** (*primary*) Body weight^c^ Fat mass—DEXA
Kanat et al.^53^, ^a^	2013	69	RCT	8	Any malignancy	Megestrol acetate + meloxicam or megestrol acetate + EPA‐enriched nutritional supplement (pharmacological)	Meloxicam + EPA‐enriched nutritional supplement	Body weight^c^ (*primary*) Lean body mass—BIA (*primary*) BMI
Poulsen et al.^54^	2013	61	RCT	5	Oesophageal/gastric/gynaecological	Nutritional counselling (nutritional)	Standard care	**Body weight** (loss vs. maintenance) (*primary*) Fat mass—BIA Fat‐free mass—BIA
Bourdel‐Marchasson et al.^55^	2014	336	RCT	10	Other mixed	Nutritional counselling (nutritional)	Standard care	Body weight^c^
Pottel et al.^56^	2014	85	Exploratory RCT	8	Head and neck	Echium oil (nutritional)	Sunflower oil	Body weight^d^ (*primary*) Lean body mass—DEXA Lean body mass—BIA Fat mass—DEXA Fat mass—BIA Fat‐free mass—BIA
Focan et al.^57^, ^a^	2015	53	RCT	7	Any malignancy	Dietetic and psychological mindfulness workshops (multi‐modal)	Standard care	**BMI** (*primary*) **Body weight** ^c^
Kapoor et al.^60^, ^a^	2016	63	RCT	8	Any malignancy	Improved atta (nutritional supplement) + nutritional counselling (nutritional)	Nutritional counselling	Body weight^c^ Mid‐arm circumference Various skinfold thickness
Mehrzad et al.^61^, ^a^	2016	70	RCT	8	Any malignancy (not brain)	Pentoxifylline (pharmacological)	Placebo	Body weight^c^ Mid‐arm circumference
Stewart Coats et al.^62^	2016	87	Phase II RCT	10	NSCLC/colorectal	Espindolol 10 mg or espindolol 2.5 mg (pharmacological)	Placebo	**Body weight** ^c^ ^,^ ^d^ (*primary*) **Lean body mass—DEXA** Fat mass—DEXA
Takayama et al.^63^	2016	181	Phase II RCT	8	NSCLC	Anamorelin 100 mg or anamorelin 50 mg (pharmacological)	Placebo	**Lean body mass—DEXA** (*primary*) Lean body mass—BIA **Fat mass—DEXA** **Fat mass—BIA** **Body weight** ^c^
Temel et al.^11^	2016	979	Phase III RCT	8	NSCLC	Anamorelin (pharmacological)	Placebo	**Lean body mass—DEXA** (*primary*) **Body weight** ^c^ **Total body mass—DEXA** **Fat mass—DEXA** **Appendicular LBM—DEXA**
Woo et al.^64^	2016	67	Phase II RCT	9	Pancreatic	Pancreatic exocrine replacement therapy (nutritional)	Placebo	Body weight^c^ ^,^ ^d^ (*primary*)
Currow et al.^65^	2017	513	Phase III RCT	8	NSCLC	Anamorelin (pharmacological)	Placebo	**Body weight** ^c^
Jatoi et al.^66^	2017	300	RCT	8	Any malignancy (not primary brain tumour)	Creatine monohydrate (nutritional)	Placebo	Body weight^d^ (*primary*)
Leedo et al.^67^	2017	40	RCT	8	Lung	Home meal delivery (nutritional)	Standard care	Body weight^c^
Sandmael et al.^68^	2017	41	Pilot RCT	9	Head and neck	Exercise and nutrition intervention during radiotherapy treatment (multi‐modal)	Exercise and nutrition intervention after radiotherapy treatment	Skeletal muscle index—CT Body weight^c^
Solheim et al.^69^	2017	46	Phase II RCT	8	NSCLC/pancreatic	Exercise, celecoxib + nutritional supplements (multi‐modal)	Standard care	**Body weight** ^c^ ^,^ ^d^ Skeletal muscle area—CT
Werner et al.^70^	2017	60	RCT	7	Pancreatic	Fish oil (nutritional)	Marine phospholipids	Body weight^d^ BMI
Katakami et al.^73^	2018	174	Phase III RCT	8	NSCLC	Anamorelin (pharmacological)	Placebo	**Lean body mass—DEXA** (*primary*) **Body weight** ^c^
Kouchaki et al.^74^	2018	90	Phase III RCT	8	Gastrointestinal	Megestrol acetate + celecoxib (pharmacological)	Megestrol acetate + placebo	Body weight^c^ ^,^ ^d^ (*primary*)
Schink et al.^75^	2018	131	Pilot non‐randomized trial	9	Any solid tumour	Whole‐body electromyostimulation + nutritional counselling (multi‐modal)	Nutritional counselling	**Lean body mass—BIA** (*primary*) Fat mass—BIA **Body weight** ^c^
Uster et al.^76^	2018	58	RCT	9	Gastrointestinal/lung	Exercise programme + nutritional counselling (multi‐modal)	Standard care	Body weight^c^
Xie et al.^77^, ^a^	2018	54	RCT	8	Lung	Thalidomide + cinobufagin (pharmacological)	Cinobufagin	Body weight^c^ Mid‐arm circumference
Britton et al.^79^	2019	307	RCT	7	Head and neck	Psychological nutritional intervention (nutritional)	Standard care	**Body weight** ^d^
Cereda et al.^80^	2019	166	RCT	8	Other mixed	Whey protein isolate supplement + nutritional counselling (nutritional)	Nutritional counselling	**Fat‐free mass index—BIA** **Body weight** ^c^
Laviano et al.^81^	2019	55	Pilot RCT	8	NSCLC	Targeted medical nutrition supplement (nutritional)	Isocaloric comparator drink	Skeletal muscle area—CT Visceral fat area—CT Appendicular LBM—DEXA Fat mass—DEXA Body weight^c^
Wiskemann et al.^84^	2019	65	RCT	5	Pancreatic	Supervised resistance training or home‐based resistance training (exercise)	Standard care	Body weight^d^ (*primary*)
Boulec et al.^85^	2020	111	RCT	7	Any malignancy	Parenteral nutrition (nutritional)	Oral feeding	Body weight^c^
Huang et al.^86^	2020	119	RCT	7	Nasopharyngeal	Nutritional supplements (nutritional)	Standard care	Body weight^c^
Movahed et al.^88^	2020	100	RCT	8	Oesophageal	Supplements ± enteral or parenteral nutrition ± pharmacotherapy + nutritional counselling (multi‐modal)	Nutritional counselling	Fat mass—BIA Fat‐free mass index—BIA Body weight^c^ BMI
Currow et al.^91^	2021	190	Phase III RCT	6	Any malignancy	Megestrol acetate or dexamethasone (pharmacological)	Placebo	Body weight^c^
Hunter et al.^92^	2021	120	Phase III RCT	7	Any solid tumour	Mirtazapine (pharmacological)	Placebo	Lean body mass—BIA Body weight^c^
Tobberup et al.^94^	2021	120	Non‐randomized trial	9	NSCLC	Fish oil + nutritional counselling + exercise programme (multi‐modal)	Standard care (historical comparator)	Skeletal muscle area—CT Body weight^c^
BMI								
Gonçalves Dias et al.^37^, ^a^	2005	64	Non‐randomized trial	1	Head and neck	Home enteral (nasogastric) feeding or oral diet + nutritional supplements (nutritional)	Oral diet	Body weight^c^ BMI Mid‐arm circumference Mid‐arm muscle area Triceps skinfold thickness
Kraft et al.^46^	2012	72	RCT	10	Pancreatic	l‐Carnitine supplement (nutritional)	Placebo	**Fat mass—BIA** **BMI**
Silander et al.^49^, ^a^	2012	134	RCT	6	Head and neck	Prophylactic PEG (nutritional)	Standard care	Body weight^d^ (*primary*) BMI
Kanat et al.^53^, ^a^	2013	69	RCT	8	Any malignancy	Megestrol acetate + meloxicam or megestrol acetate + EPA‐enriched nutritional supplement (pharmacological)	Meloxicam + EPA‐enriched nutritional supplement	Body weight^c^ (*primary*) Lean body mass—BIA (*primary*) BMI
Focan et al.^57^, ^a^	2015	53	RCT	7	Any malignancy	Dietetic and psychological mindfulness workshops (multi‐modal)	Standard care	**BMI** (*primary*) **Body weight** ^c^
Capozzi et al.^59^	2016	60	Exploratory RCT	8	Head and neck	Early ‘lifestyle intervention’ (individualized exercise with education and support) (exercise)	Delayed ‘lifestyle intervention’ (individualized exercise with education and support)	Lean body mass—DEXA Fat mass—DEXA BMI
Werner et al.^70^	2017	60	RCT	7	Pancreatic	Fish oil (nutritional)	Marine phospholipids	Body weight^d^ BMI
Ziętarska et al.^71^	2017	95	RCT	6	Colorectal	Nutritional supplements (nutritional)	Standard care	BMI
Akita et al.^78^	2019	62	RCT	8	Pancreatic	EPA‐enriched nutritional supplement (nutritional)	Standard care	Lean body mass—BIA Fat mass—BIA Psoas muscle area—CT BMI
Movahed et al.^88^	2020	100	RCT	8	Oesophageal	Supplements ± enteral or parenteral nutrition ± pharmacotherapy + nutritional counselling (multi‐modal)	Nutritional counselling	Fat mass—BIA Fat‐free mass index—BIA Body weight^c^ BMI
Qiu et al.^89^	2020	96	RCT	6	Oesophageal	Nutritional counselling (nutritional)	Standard care	BMI
Storck et al.^90^	2020	52	RCT	10	Other mixed	Protein supplement + nutritional counselling + exercise programme (multi‐modal)	Standard care	Lean body mass—BIA Fat mass—BIA BMI
Kutz et al.^93^	2021	58	RCT	7	Head and neck	Nutritional counselling (nutritional)	Standard care	BMI Fat‐free mass—BIA
Other anthropometry								
Downer et al.^15^, ^a^	1993	60	RCT	1	Any malignancy	Medroxyprogesterone acetate (pharmacological)	Placebo	Body weight^c^ (*primary*) Mid‐arm circumference Triceps skinfold thickness
Ovesen et al.^17^, ^a^	1993	105	RCT	8	Small‐cell‐lung/ovarian/breast	Nutritional counselling (nutritional)	Standard care	Body weight^c^ (*primary*) Arm muscle area **Triceps skinfold thickness**
Beller et al.^22^, ^a^	1997	240	RCT	4	Any malignancy (not hormone dependent)	Megestrol acetate 480 mg or megestrol acetate 160 mg (pharmacological)	Placebo	Body weight^c^ (*primary*) Mid‐arm circumference Mid‐arm fat area Mid‐arm muscle area Triceps skinfold thickness
Vadell et al.^26^, ^a^	1998	150	RCT	5	Any malignancy	Megestrol acetate 480 mg or megestrol acetate 160 mg (pharmacological)	Placebo	**Body weight** ^c^ (*primary*) Mid‐arm circumference **Triceps skinfold thickness**
McMillan et al.^28^, ^a^	1999	73	RCT	7	Gastrointestinal	Megestrol acetate + ibuprofen (pharmacological)	Megestrol acetate + placebo	**Body weight** ^c^ (*primary*) **Mid‐arm circumference** Triceps skinfold thickness Biceps skinfold thickness
Bruera et al.^33^, ^a^	2003	91	RCT	7	Any malignancy	Fish oil capsules (nutritional)	Placebo	Lean body mass—BIA Body weight^c^ Mid‐arm muscle circumference Triceps skinfold thickness Subscapular skinfold thickness
Gonçalves Dias et al.^37^, ^a^	2005	64	Non‐randomized trial	1	Head and neck	Home enteral (nasogastric) feeding or oral diet + nutritional supplements (nutritional)	Oral diet	Body weight^c^ BMI Mid‐arm circumference Mid‐arm muscle area Triceps skinfold thickness
Lundholm et al.^36^, ^a^	2004	309	RCT	5	Any solid tumour	Indomethacin + erythropoietin + nutritional support + home total parenteral nutrition (multi‐modal)	Indomethacin + erythropoietin	Fat mass—DEXA Lean body mass—DEXA Body weight^c^ Mid‐arm muscle circumference Triceps skinfold thickness
Gordon et al.^38^, ^a^	2005	50	RCT	10	Pancreatic	Thalidomide (pharmacological)	Placebo	**Body weight** ^c^ (*primary*) **Bone‐free arm muscle area**
Berk et al.^40^, ^a^	2008	472	RCT	9	Any solid tumour	Nutritional supplement (nutritional)	Placebo	Lean body mass—BIA (*primary*) Body weight^d^ Various skinfold thickness
Beijer et al.^42^, ^a^	2009	100	RCT	8	Any malignancy	Adenosine 5′‐triphosphate (pharmacological)	Standard care	**Triceps skinfold thickness** (*primary*) Body weight^c^ Mid‐arm circumference
Kapoor et al.^60^, ^a^	2016	63	RCT	8	Any malignancy	Improved atta (nutritional supplement) + nutritional counselling (nutritional)	Nutritional counselling	Body weight^c^ Mid‐arm circumference Various skinfold thickness
Mehrzad et al.^61^, ^a^	2016	70	RCT	8	Any malignancy (not brain)	Pentoxifylline (pharmacological)	Placebo	Body weight^c^ Mid‐arm circumference
Xie et al.^77^, ^a^	2018	54	RCT	8	Lung	Thalidomide + cinobufagin (pharmacological)	Cinobufagin	Body weight^c^ Mid‐arm circumference

*Note*: Sample sizes are reported as per ‘intention to treat’. Abbreviations: BIA, bioimpedance analysis; BMI, body mass index; CT, computed tomography; DEXA, dual‐energy X‐ray absorptiometry; EPA, eicosapentaenoic acid; NSCLC, non‐small‐cell lung cancer; PEG, percutaneous endoscopic gastrostomy; RCT, randomized controlled trial.

^a^ Considered more than one anthropometric estimate of body composition.

^b^ Endpoints that are bold underlined had a statistically significant difference between groups.

^c^ Endpoint expressed as change in absolute value from baseline.

^d^ Endpoint expressed as percentage change from baseline.

## Appendix C Included studies considering bioelectrical or radiological assessments of body composition

**Table:**

Author (reference)	Year	Sample size	Study design	Quality score	Primary cancer site	Intervention	Comparator	Body composition outcomes^b^
BIA body composition								
Bruera et al.^33^	2003	91	RCT	7	Any malignancy	Fish oil capsules (nutritional)	Placebo	Lean body mass—BIA Body weight Mid‐arm muscle circumference Triceps skinfold thickness Subscapular skinfold thickness
Fearon et al.^34^	2003	200	RCT	8	Pancreatic	*n*‐3 fatty acid and antioxidant‐enriched supplement (nutritional)	Supplement without *n*‐3 fatty acid and antioxidants	Lean body mass—BIA Body weight
Isenring et al.^35^	2004	60	RCT	8	Gastrointestinal/head and neck	Nutrition counselling and protocol (nutritional)	Standard care	Fat‐free mass—BIA **Body weight**
Fearon et al.^39^	2006	518	RCT	8	Gastrointestinal/lung	EPA 2 g or EPA 4 g (pharmacological)	Placebo	Lean body mass—BIA Body weight
Berk et al.^40^	2008	472	RCT	9	Any solid tumour	Nutritional supplement (nutritional)	Placebo	Lean body mass—BIA (*primary*) Body weight Various skinfold thickness
Wiedenmann et al.^41^	2008	86	Phase II RCT	7	Pancreatic	Infliximab 5 mg/kg or infliximab 3 mg/kg (pharmacological)	Placebo	Lean body mass—BIA (*primary*)
Mantovani et al.^43^, ^a^	2010	332	Phase III RCT	7	Any malignancy	Megestrol acetate or EPA‐enriched nutritional supplement or l‐carnitine or thalidomide (pharmacological)	Megestrol acetate + EPA‐enriched nutritional supplement + l‐carnitine + thalidomide	**Lean body mass—DEXA** (*primary*) Lean body mass—BIA (*primary*) **Lean body mass—CT** (*primary*)
Kraft et al.^46^	2012	72	RCT	10	Pancreatic	l‐Carnitine supplement (nutritional)	Placebo	**Fat mass—BIA** **BMI**
Madeddu et al.^48^, ^a^	2012	60	Phase III RCT	7	Any malignancy	l‐Carnitine + celecoxib + megestrol acetate (pharmacological)	l‐Carnitine + celecoxib	Lean body mass—DEXA (*primary*) Lean body mass—CT (*primary*) Lean body mass—BIA (*primary*)
Del Fabbro et al.^51^	2013	73	RCT	10	Gastrointestinal/lung	Melatonin (pharmacological)	Placebo	Body weight (*primary*) Lean body mass—BIA Fat‐free mass—BIA
Kanat et al.^53^	2013	69	RCT	8	Any malignancy	Megestrol acetate + meloxicam or megestrol acetate + EPA‐enriched nutritional supplement (pharmacological)	Meloxicam + EPA‐enriched nutritional supplement	Body weight (*primary*) Lean body mass—BIA (*primary*) BMI
Poulsen et al.^54^	2013	61	RCT	5	Oesophageal/gastric/gynaecological	Nutritional counselling (nutritional)	Standard care	**Body weight** (*primary*) Fat mass—BIA Fat‐free mass—BIA
Pottel et al.^56^, ^a^	2014	85	Exploratory RCT	8	Head and neck	Echium oil (nutritional)	Sunflower oil	Body weight (*primary*) Lean body mass—DEXA Fat mass—DEXA Lean body mass—BIA Fat mass—BIA Fat‐free mass—BIA
Takayama et al.^63^, ^a^	2016	181	Phase II RCT	8	NSCLC	Anamorelin 100 mg or anamorelin 50 mg (pharmacological)	Placebo	**Lean body mass—DEXA** (*primary*) **Fat mass—DEXA** Lean body mass—BIA **Fat mass—BIA** **Body weight**
Schink et al.^75^	2018	131	Pilot non‐randomized trial	9	Any solid tumour	Whole‐body electromyostimulation + nutritional counselling (multi‐modal)	Nutritional counselling	**Lean body mass—BIA** (*primary*) Fat mass—BIA **Body weight**
Akita et al.^78^, ^a^	2019	62	RCT	8	Pancreatic	EPA‐enriched nutritional supplement + nutritional guidance (nutritional)	Nutritional guidance	Lean body mass—BIA Fat mass—BIA Psoas muscle area—CT BMI
Cereda et al.^80^	2019	166	RCT	8	Other mixed	Whey protein isolate supplement + nutritional counselling (nutritional)	Nutritional counselling	**Fat‐free mass index—BIA** **Body weight**
Obling et al.^82^	2019	47	RCT	7	Gastrointestinal	Supplemental home parenteral nutrition and nutritional counselling (nutritional)	Nutritional counselling	**Fat‐free mass—BIA** (*primary*) **Fat‐free mass index—BIA** (*primary*)
Stuecher et al.^83^	2019	44	RCT	8	Gastrointestinal	Walking exercise programme (exercise)	Standard care	**Lean body mass—BIA**
Movahed et al.^88^	2020	100	RCT	8	Oesophageal	Supplements ± enteral or parenteral nutrition ± pharmacotherapy + nutritional counselling (multi‐modal)	Nutritional counselling	Fat mass—BIA Fat‐free mass index—BIA Body weight BMI
Storck et al.^90^	2020	52	RCT	10	Other mixed	Whey protein supplement + nutritional counselling + exercise programme (multi‐modal)	Standard care	Lean body mass—BIA Fat mass—BIA BMI
Hunter et al.^92^	2021	120	Phase III RCT	7	Any solid tumour	Mirtazapine (pharmacological)	Placebo	Lean body mass—BIA Body weight
Kutz et al.^93^	2021	58	RCT	7	Head and neck	Nutritional counselling (nutritional)	Standard care	BMI Fat‐free mass—BIA
DEXA body composition								
Daneryd et al.^24^	1998	180	RCT	7	Any malignancy	Indomethacin + erythropoietin (pharmacological)	Indomethacin	Lean body mass—DEXA **Body weight**
Lundholm et al.^36^	2004	309	RCT	5	Any solid tumour	Indomethacin + erythropoietin + nutritional support + home total parenteral nutrition (multi‐modal)	Indomethacin + erythropoietin	Fat mass—DEXA Lean body mass—DEXA Body weight Mid‐arm muscle circumference Triceps skinfold thickness
Mantovani et al.^43^, ^a^	2010	332	Phase II RCT	9	Any malignancy	Megestrol acetate or EPA‐enriched nutritional supplement or l‐carnitine or thalidomide (pharmacological)	Megestrol acetate + EPA‐enriched nutritional supplement + l‐carnitine + thalidomide	**Lean body mass—DEXA** (*primary*) Lean body mass—BIA (*primary*) **Lean body mass—CT** (*primary*)
Macciò et al.^47^	2012	144	Phase III RCT	8	Gynaecological	Megestrol acetate + l‐carnitine + celecoxib + antioxidants (pharmacological)	Megestrol acetate	**Lean body mass—DEXA** (*primary*)
Madeddu et al.^48^, ^a^	2012	60	Phase III RCT	7	Any malignancy	l‐Carnitine + celecoxib + megestrol acetate (pharmacological)	l‐Carnitine + celecoxib	Lean body mass—DEXA (*primary*) Lean body mass—CT (*primary*) Lean body mass—BIA (*primary*)
Dobs et al.^52^	2013	159	Phase II RCT	8	Other mixed	Enobosarm 1 mg or enobosarm 3 mg (pharmacological)	Placebo	**Lean body mass—DEXA** (*primary*) Body weight Fat mass—DEXA
Pottel et al.^56^, ^a^	2014	85	Exploratory RCT	8	Head and neck	Echium oil (nutritional)	Sunflower oil	Body weight (*primary*) Lean body mass—DEXA Lean body mass—BIA Fat mass—DEXA Fat mass—BIA Fat‐free mass—BIA
Garcia et al.^58^	2015	82	Phase II RCT	7	Any malignancy	Anamorelin 50 mg (pharmacological)	Placebo	**Lean body mass—DEXA** (*primary*) **Appendicular LBM—DEXA** **Total body mass—DEXA** Fat mass—DEXA
Capozzi et al.^59^	2016	60	Exploratory RCT	8	Head and neck	Early ‘lifestyle intervention’ (individualized exercise with education and support) (exercise)	Delayed ‘lifestyle intervention’ (individualized exercise with education and support)	Lean body mass—DEXA Fat mass—DEXA BMI
Stewart Coats et al.^62^	2016	87	Phase II RCT	10	NSCLC/colorectal	Espindolol 10 mg or espindolol 2.5 mg (pharmacological)	Placebo	**Body weight** (*primary*) **Lean body mass—DEXA** Fat mass—DEXA
Takayama et al.^63^, ^a^	2016	181	Phase II RCT	8	NSCLC	Anamorelin 100 mg or anamorelin 50 mg (pharmacological)	Placebo	**Lean body mass—DEXA** (*primary*) **Fat mass—DEXA** Lean body mass—BIA **Fat mass—BIA** **Body weight**
Temel et al.^11^	2016	979	Phase III RCT	8	NSCLC	Anamorelin (pharmacological)	Placebo	**Lean body mass—DEXA** (*primary*) **Body weight** **Total body mass—DEXA** **Fat mass—DEXA** **Appendicular LBM—DEXA**
Katakami et al.^73^	2018	174	Phase III RCT	8	NSCLC	Anamorelin (pharmacological)	Placebo	**Lean body mass—DEXA** (*primary*) **Body weight**
Golan et al.^72^, ^a^	2018	125	Phase II RCT	7	Pancreatic	Anti‐myostatin antibody 300 mg or anti‐myostatin antibody 100 mg (pharmacological)	Placebo	Thigh muscle volume—CT Skeletal muscle area—CT Adipose tissue area—CT Lean body mass—DEXA Fat mass—DEXA
Laviano et al.^81^, ^a^	2019	55	Pilot RCT	8	NSCLC	Targeted medical nutrition supplement (nutritional)	Isocaloric comparator drink	Skeletal muscle area—CT Visceral fat area—CT Appendicular LBM—DEXA Fat mass—DEXA Body weight
Kamel et al.^87^	2020	40	RCT	7	Pancreatic	Resistance training (exercise)	Standard care	**Appendicular LBM—DEXA** **Fat mass—DEXA**
CT body composition								
Mantovani et al.^43^, ^a^	2010	332	Phase III RCT	7	Any malignancy	Megestrol acetate or EPA‐enriched nutritional supplement or l‐carnitine or thalidomide (pharmacological)	Megestrol acetate + EPA‐enriched nutritional supplement + l‐carnitine + thalidomide	**Lean body mass—DEXA** (*primary*) Lean body mass—BIA (*primary*) **Lean body mass—CT** (*primary*)
Madeddu et al.^48^, ^a^	2012	60	Phase III RCT	7	Any malignancy	l‐Carnitine + celecoxib + megestrol acetate (pharmacological)	l‐Carnitine + celecoxib	Lean body mass—DEXA (*primary*) Lean body mass—CT (*primary*) Lean body mass—BIA (*primary*)
Sandmael et al.^68^	2017	41	Pilot RCT	9	Head and neck	Exercise and nutrition intervention during radiotherapy treatment (multi‐modal)	Exercise and nutrition intervention after radiotherapy treatment	Skeletal muscle index—CT Body weight
Solheim et al.^69^	2017	46	Phase II RCT	8	NSCLC/pancreatic	Exercise, celecoxib + nutritional supplements (multi‐modal)	Standard care	**Body weight** Skeletal muscle area—CT
Golan et al.^72^, ^a^	2018	125	Phase II RCT	7	Pancreatic	Anti‐myostatin antibody 300 mg or anti‐myostatin antibody 100 mg (pharmacological)	Placebo	Thigh muscle volume—CT Skeletal muscle area—CT Adipose tissue area—CT Lean body mass—DEXA Fat mass—DEXA
Akita et al.^78^, ^a^	2019	62	RCT	8	Pancreatic	EPA‐enriched nutritional supplement + nutritional guidance (nutritional)	Nutritional guidance	Lean body mass—BIA Fat mass—BIA Psoas muscle area—CT BMI
Laviano et al.^81^, ^a^	2019	55	Pilot RCT	8	NSCLC	Targeted medical nutrition supplement (nutritional)	Isocaloric comparator drink	Skeletal muscle area—CT Visceral fat area—CT Appendicular LBM—DEXA Fat mass—DEXA Body weight
Tobberup et al.^94^	2021	120	Non‐randomized trial	9	NSCLC	Fish oil + nutritional counselling + exercise programme (multi‐modal)	Standard care (historical comparator)	Skeletal muscle area—CT Body weight

*Note*: Sample sizes are reported as per ‘intention to treat’.

Abbreviations: BIA, bioimpedance analysis; BMI, body mass index; CT, computed tomography; DEXA, dual‐energy X‐ray absorptiometry; EPA, eicosapentaenoic acid; LBM, lean body mass; NSCLC, non‐small‐cell lung cancer; RCT, randomized controlled trial.

^a^ Considered more than one radiological or bioelectrical estimate of body composition.

^b^ Endpoints that are bold underlined had a statistically significant difference between groups.
